# Electroconvulsive therapy triggers a reversible decrease in brain N-acetylaspartate

**DOI:** 10.3389/fpsyt.2023.1155689

**Published:** 2023-06-09

**Authors:** Vera J. Erchinger, Alexander R. Craven, Lars Ersland, Ketil J. Oedegaard, Christoffer A. Bartz-Johannessen, Åsa Hammar, Jan Haavik, Frank Riemer, Ute Kessler, Leif Oltedal

**Affiliations:** ^1^Department of Clinical Medicine, University of Bergen, Bergen, Norway; ^2^Mohn Medical Imaging and Visualization Centre, Department of Radiology, Haukeland University Hospital, Bergen, Norway; ^3^Department of Biological and Medical Psychology, University of Bergen, Bergen, Norway; ^4^NORMENT—Norwegian Centre for Mental Disorders Research, University of Bergen, Bergen, Norway; ^5^Department of Clinical Engineering, Haukeland University Hospital, Bergen, Norway; ^6^Division of Psychiatry, Haukeland University Hospital, Bergen, Norway; ^7^Department of Biomedicine, University of Bergen, Bergen, Norway

**Keywords:** depression, electroconvulsive therapy, ^1^H-MRS nuclear magnetic resonance spectroscopy, neurometabolites, choline, myoinositol, glutamate

## Abstract

**Introduction:**

Based on previous research on electroconvulsive therapy (ECT) we have proposed a model where *disruption, potentiation,* and *rewiring of brain networks* occur in sequence and serve as the underlying therapeutic mechanism of ECT. This model implies that a temporary disturbance of neuronal networks (disruption) is followed by a trophic effect (potentiation), which enables the rewiring of neuronal circuits to a more euthymic functioning brain. We hypothesized that disruption of neuronal networks could trigger biochemical alterations leading to a temporary decrease in N-acetylaspartate (tNAA, considered a marker of neuronal integrity), while choline (a membrane component), myo-Inositol (mI, astroglia marker), and glutamate/glutamine (Glx, excitatory neurotransmitter) were postulated to increase. Previous magnetic resonance spectroscopy studies, reporting diverse findings, have used two different referencing methods - creatine ratios and tissue corrected values referenced to water – for the quantification of brain metabolites. Changes in creatine during ECT have also been reported, which may confound estimates adopting this as an internal reference.

**Methods:**

Using MR spectroscopy, we investigated 31 moderately to severely depressed patients and 19 healthy controls before, during, and after ECT or at similar time points (for controls). We tested whether biochemical alterations in tNAA, choline, mI, and Glx lend support to the *disrupt, potentiate, and rewire hypothesis.* We used both creatine ratios and water-scaled values for the quantification of brain metabolites to validate the results across referencing methods.

**Results:**

Levels of tNAA in the anterior cingulate cortex decreased after an ECT treatment series (average 10.6 sessions) by 6% (*p* = 0.007, creatine ratio) and 3% (*p* = 0.02, water referenced) but returned to baseline 6 months after ECT. Compared to after treatment series tNAA levels at 6-month follow-up had increased in both creatine ratio (+6%, *p* < 0.001) and water referenced data (+7%, *p* < 0.001). Findings for other brain metabolites varied and could not be validated across referencing methods.

**Discussion:**

Our findings suggest that prior research must be interpreted with care, as several referencing and processing methods have been used in the past. Yet, the results for tNAA were robust across quantification methods and concur with relevant parts of the *disrupt, potentiate, and rewire* model.

## 1. Introduction

Electroconvulsive therapy (ECT) is a therapy for depression that is mainly used in non-responders to antidepressant pharmacotherapy and in patients requiring fast and effective symptom alleviation (1). The treatment is performed by placing electrodes on the patient’s scalp and applying an electrical current to the brain, inducing a seizure. Although it is well established that ECT is an effective treatment for major depressive disorder (MDD) (2) the neurobiological underpinnings of the clinical response are still being investigated.

MR spectroscopy (MRS) is a practical and non-invasive MR-technique that allows investigation of the brain’s neurobiology *in vivo*. By exploiting the differences in resonance frequency between molecules certain metabolites can be studied. This gives a unique opportunity to study the neurobiological underpinnings of ECT. Most commonly the hydrogen nucleus (a single proton) is used as origin for the MRS signal, giving the ^1^H-MRS spectrum. The total received signal, hence the estimated amplitude and area under the curve, will depend on several factors, including field strength, relaxation effects, and coil properties and loading; several of these are difficult to reliably control for. As such, a stable reference signal is commonly adopted to scale the amplitude and correct for these unknown factors. Though processing pipelines vary, two main referencing methods are used: the metabolite ratio relative to total creatine (/tCr), or water referenced values (/H_2_O). Both have been used in previous ECT research (3). Since variation in creatine itself has been shown to occur following ECT (4) we have explored both creatine ratios and water referenced metabolite levels. When examining neurobiological underpinnings of ECT, several molecules are of interest - such as N-acetylaspartate (NAA), choline (Cho), myo-Inositol (mI), and glutamate/glutamine (Glx).

The disrupt, potentiate, and rewire (DPR) hypothesis (5) suggests that ECT leads to temporary disruption of neuronal networks, followed by a trophic effect (potentiation), which enables the rewiring of neuronal circuits to a more euthymic functioning brain. It is assumed that in the depressed state, before ECT, the brain has a low plastic potential [as shown in both animal and post-mortem studies, summarized by Ousdal (5)], and it is hypothesized that the temporary disruption created by ECT clinically is seen as post-ictal confusion and, for some, as reduced cognitive performance. This is supported by a meta-analysis which found reduced cognitive functioning 4 days after ECT, but a return to baseline levels or better was seen after 15 days (6). On a neuroradiological level, we hypothesize that disruption is seen as metabolite alterations (3), altered functional connectivity (7), and changed white matter integrity (8). Responding to this disruption, temporary upregulation of neuroplasticity is seen (“potentiate”), reflected in both metabolite changes and increased gray matter volume. During or following this neuroplastic upregulation, previously maladaptive depressive networks may rewire to non-depressed states. Although MRS cannot test the complete DPR-hypothesis, we explored whether metabolite levels measured over the ECT treatment course are as expected under the framework of the DPR- hypothesis.

NAA is the most abundant metabolite in the ^1^H-MRS spectrum of the healthy brain. Decreased levels of NAA are seen in brain injury and disease, and in ^1^H-MRS, NAA is considered a marker of neuronal integrity (9, 10). ^1^H-MRS *total* NAA values (tNAA) are comprised of NAA and closely resonating NAAG, which only amounts to a small part of the signal intensity (10). Maddock and Buonocre have summarized findings for depression, where lower NAA levels have been seen in bipolar depression compared to controls, but not in unipolar depression (11). Several studies have also found lower NAA levels after ECT treatment, as summarized in a recent review (3). NAA could serve as a potential marker of the temporary disruption in the disrupt-potentiate and rewire hypothesis. Equivalent to this theory, NAA decrease has been seen to reverse after successful treatment in epilepsy (12).

Choline is primarily a building block for cell membranes. The choline peak reported in ^1^H-MRS at 3.2 ppm consists of several choline compounds: glycerophosphocholine (GPC), phosphocholine (PCh), and free choline, often reported together as total choline (tCho). An increase in choline may reflect both choline synthesis and membrane damage, hence it must be interpreted with care (13). Reviews report increased levels of choline in depression, primarily in the basal ganglia (11, 14), and attribute this to increased membrane turnover. In both a review of depression (15), and an ECT specific review (3), an increase in choline has also been found comparing pre-treatment to post-treatment values. In relation to the DPR-hypothesis, an increase in choline could reflect both disruption (increase due to affection of cell membranes) as well as potentiation and rewiring (increase due to increased membrane turnover).

Primarily three roles are known for mI: as a lipid component for biomembranes, part of an intracellular second messenger system (releasing calcium), and as an osmolyte (10). The antidepressant and mood stabilizer lithium affects Ins levels by blocking its resynthesis. It has therefore been hypothesized that high Ins levels are part of the pathogenesis in bipolar disorder (16, 17), but no consistent Ins alterations have been shown in bipolar patients (11). In depressed patients, ^1^H-MRS investigations have not shown higher levels of Ins compared to controls, but rather decreased levels (18–20), which increase with pharmacotherapy (18). Additionally, orally administered Ins has been studied as a potential treatment for depression (21), and a meta-analysis (22) suggested that depressed patients might benefit from Ins. In ^1^H-MRS, mI is proposed as a marker for glia cell proliferation (23), and is widely studied as such (16), though this interpretation is disputed - as neural cell lines also have displayed high levels of mI (24). In light of the DPR hypothesis, an increase in mI could reflect the rewiring and potentiation, due to glial cell proliferation after disruption. In ECT patients, one investigation has shown an increase of mI in the anterior cingulate cortex (ACC) after treatment (25).

Glutamate (Glu) is the main excitatory neurotransmitter in the brain; measured with ^1^H-MRS at 3T it is difficult to distinguish from Glutamine (Gln), hence the two are often reported together as Glx – wherein Glu is usually the dominant component due to its substantially higher concentration. Its concentration has been measured to be lower in depressed subjects compared to healthy controls (11, 14). Both neurostimulation and medication have been seen to increase Glu levels in depressed patients (11). However, excessive levels of extracellular Glx are neurotoxic (10). Within the theoretic framework of the DPR hypothesis, excessive Glu release during seizures could be a factor mediating neuronal disruption and thus a change in Glx levels would be expected to be the opposite of a change in tNAA levels.

The total Creatine (tCr) signal in ^1^H-MRS originates from Creatine (Cr) and phosphocreatine (PCr). tCr is often assumed to be somewhat stable and is therefore often chosen as an internal concentration reference. However, Cr concentration in the brain may be related to neural activity and/or vascularization and has been shown to vary with intake (26) and in certain conditions and pathologies (27–30), therefore its role as a reference metabolite has been criticized (10, 31). One previous ^1^H-MRS investigation in ECT patients has shown an increase in Cr in the ACC related to ECT (4). Both creatine ratios and water referenced metabolites have been used in previous ECT literature, but these referencing methods have not yet been compared in this clinical setting.

### 1.1. Aim and hypotheses

In this investigation, we aimed to investigate metabolite changes during ECT treatment and relate them to the DPR hypothesis. Specifically, we hypothesized that tNAA levels decrease after ECT treatment, due to temporary disruption of neuronal integrity. At 6-month follow-up, tNAA levels return to baseline levels or higher. There is a negative correlation between tNAA and everyday memory impairment. tCho levels increase after ECT due to temporary disruption of cell membranes. This increase from baseline is no longer seen at 6-month follow-up. There is a positive correlation between tCho rise and everyday memory impairment. Baseline mI levels are lower in patients compared to controls and increase during treatment. After the ECT series, and at follow-up, mI levels remain increased for responders indicating potentiation and rewiring. Glx levels at baseline are lower in patients compared to controls. Glx levels increase with ECT and remain increased at 6-month follow-up but have their peak levels after ECT treatment series, possibly as a part of the mechanism behind disruption.

## 2. Materials and methods

This study was approved by the Regional Committee for Medical and Health Research Ethics, REC South East, Norway (2013/1032). The protocol of this study has been published previously (32); here we summarize key points relevant to the analyses in the present work.

### 2.1. Study participants and assessments

Patients referred to ECT treatment at Haukeland University hospital, Norway, between September 2013 to September 2018 were asked to participate in the study. A Montgomery and Aasberg depression rating scale (33) (MADRS) score of minimum 25 and age over 18 years was required to qualify for participation. To control for time effects, age and sex-matched healthy controls (HC) were recruited from the general population in the same area as patients through advertising in public areas. HC could not have a history of psychiatric disease, and MADRS score was taken to document that HC scored below the clinical range (<7). Written informed consent was provided by all participants. The responsible clinician (for patients) or research assistant (for HC) evaluated eligibility for inclusion and the ability to give written informed consent. Subjects who were not able to give informed consent, who were pregnant, or who could not undertake the MRI investigation were excluded. Patients who had undergone ECT treatment during the last 12 months were also excluded. Throughout the treatment course, depression severity was monitored by MADRS. MADRS scores for study purpose were acquired <7 days before participation in the study, and at the same timepoint as MR examinations after treatment (TP3) and at 6-month follow-up (TP4). Remission was defined as a ≥ 50% reduction of baseline MADRS score and MADRS ≤10. The everyday memory questionnaire (EMQ-28) (34, 35) is a comprehensive, subjective evaluation of everyday memory and was used to assess subjective effects on cognition. The EMQ-28 assesses everyday memory with 28 statements of forgetfulness and their occurrence, ranging from 0 (none) to 8 (more than once a day) leading to a score of maximum 224, indicating the most severe forgetfulness.

### 2.2. ECT procedure

Right unilateral (RUL) electrode placement ECT was performed using a Thymatron System IV (Somatics LLC, Venice, FL, USA). The initial stimulus charge was calculated by an age-based algorithm, where the patient’s age in years x5 ≅ stimulus charge in mC. The stimulus was increased during the treatment series due to increase in seizure threshold. All patients were administered anesthesia (thiopental) and neuromuscular blockade (succinylcholine). All patients were hyperoxygenated before and during anesthesia.

### 2.3. MR-acquisition and data analysis

**Figure 1:**
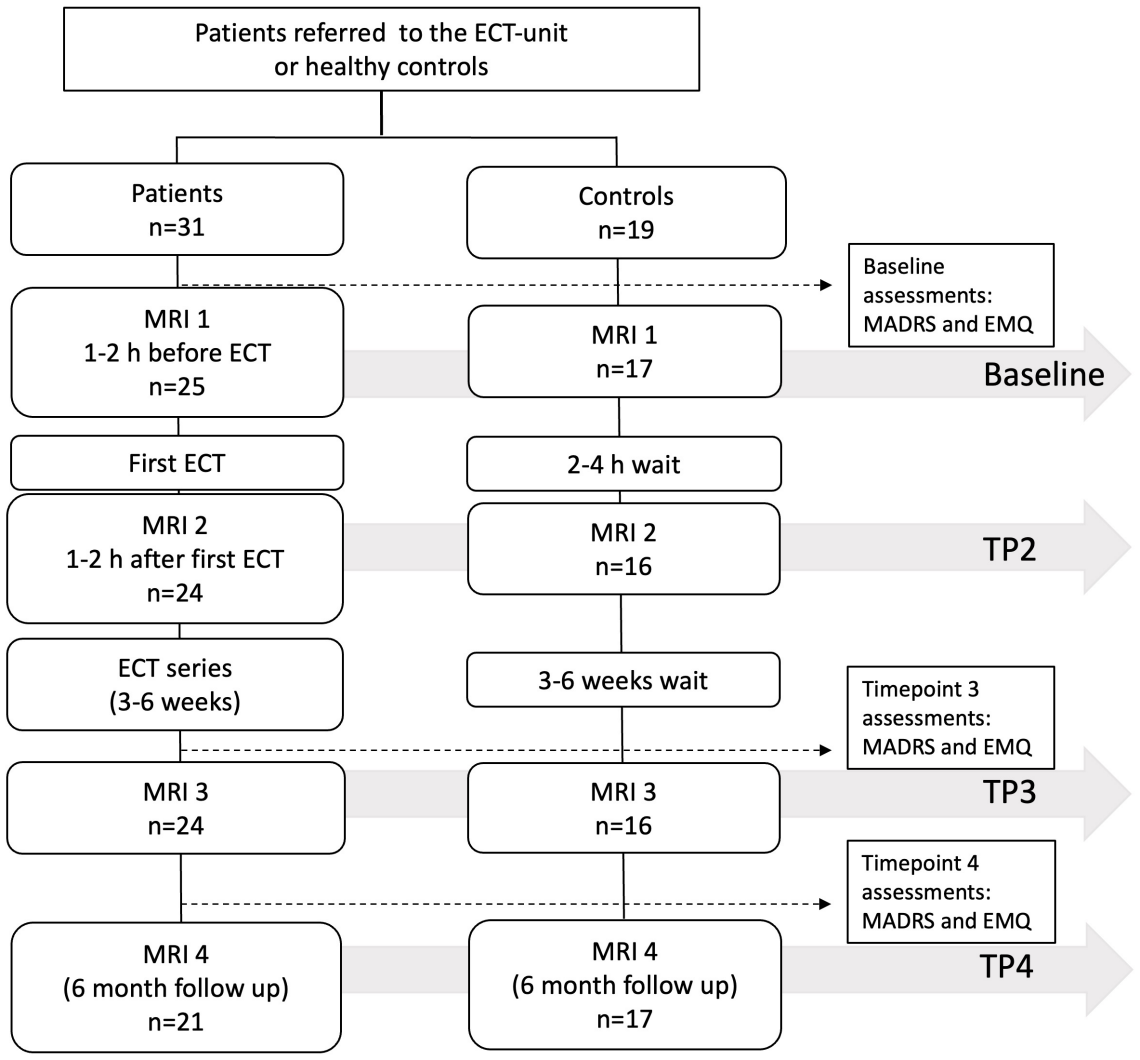
Study flowchart. Forty patients and 20 HC were enrolled in the study. Due to missing data, the number of analyzed participants at each timepoint varied as indicated in the figure.

Patients were scanned at four timepoints: 1–2 h before treatment (Baseline), 1–2 h after first treatment (TP2), 7–14 days after completion of ECT series (TP3), and 6 months after ECT series (TP4). HC were scanned at similar time intervals as patients. The study flowchart can be seen in Figure 1.

**Figure 2:**
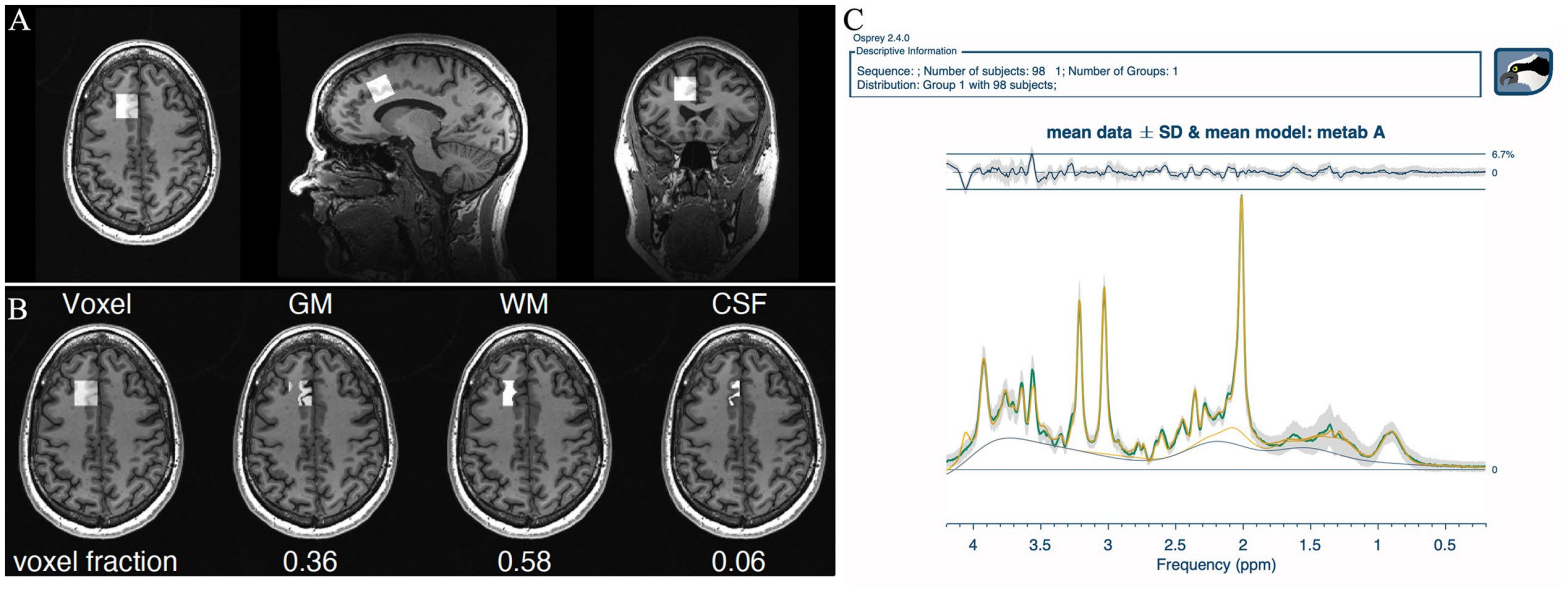
Output from Osprey (36). **(A)** Voxel placement in the ACC. **(B)** Voxel segmentation into gray matter, white matter, and CSF. **(C)** Mean spectrum of all patients at all timepoints for visualization of overall quality. Quality metrics: SNR: 48 ± 7, linewidth 5.72 ± 1.08 Hz, mean relative amplitude residual 2.86%.

Imaging and MRS were performed on a 3 Tesla GE Discovery 750 scanner system (Waukesha, WI, USA). A 32-channel head coil was used. For voxel localization, a 3D T1 weighted fast spoiled gradient echo (FSPGR) sequence was used (echo time = 2.9 ms, repetition time = 6.7 ms, inversion time = 600 ms, flip angle 8 degrees, field of view = 25.6, matrix size 256×256, giving an isotropic voxel size of 1x1x1 mm). The single voxel point resolved spectroscopy (SV-PRESS) voxel was placed in the anterior cingulate cortex (ACC), angled to follow the foremost slope of the corpus callosum (Figure 2). Voxel placement alternated between left and right side for every other patient to balance for lateralization effects, as right unilateral ECT creates an anatomically uneven electrical field and volume change (37). Voxel size was 2×2×2 cm (8 mL). Parameters for SV-PRESS were: echo time = 35 ms, repetition time = 1,500 ms, 128 scans, spectral width = 5,000 Hz, number of spectral points = 4,096 points, water suppression method: CHESS. Post-processing, voxel segmentation, and tissue correction were performed using the Osprey software version 2.4.0 (36). The acquired FSPGR acquisition was segmented into CSF, white matter, and gray matter, before adjusting the metabolite concentration to the proportion of gray and white matter in the voxel (38). Metabolite concentrations are reported in institutional units (IU) and presented both as creatine ratios and water referenced values. For further details, see the table for minimum reporting standards in MRS (39) (Supplementary material S1). All spectra were visually inspected by one reader [VJE], and aberrant spectra were judged for further consensus evaluation by one additional reader [LE or ARC]. If one or more of the metabolite concentration estimates for tNAA, tCho, Glx, mI and tCr were considered extreme outliers (>the 3rd quartile +3 interquartile ranges or < the 1st quartile – 3 interquartile ranges) spectra were flagged and inspected individually. Flagged spectra were excluded if CSF proportion (for water referenced data) seemed incorrect based on visual inspection of the FSPGR acquisition compared to the proportion given in the automatic segmentation. Figure 2 shows the mean ^1^H-MRS-spectrum for patients. Metabolite concentrations are reported as mean ± SD.

### 2.4. Statistical analyses

Statistical analyses were performed using the R statistical software, version 4.1.3 (40). All analyses were performed both on the creatine ratio and the water referenced metabolite concentration estimates. Baseline differences between groups were investigated using two-sample’s *t*-tests (for age, voxel composition, metabolite levels) or Pearson chi squared tests (for sex).

For longitudinal analyses of patients’ ^1^H-MRS data the nlme package (41) was used to perform linear mixed effects analyses. Timepoint, sex, age, number of ECT treatments and remission were entered as fixed effects, while participant ID was entered as random effect. The contrast package (42) was used for time-specific comparisons.

Treatment effect and side effect, monitored using MADRS and EMQ-28 respectively, were explored with linear models, comparing delta change between baseline and after treatment. Sex and age were set as fixed effects. Correction for multiple testing was not performed.

## 3. Results

### 3.1. Study group characteristics and participation

Forty patients and 20 HC participated in the study. Due to a scanner update and missing MR data the total number of participants analyzed in this paper was 31 patients and 19 HC, but not all participants had data from all timepoints. This resulted in 25 patients (17 HC) at baseline, 24 patients (16 HC) at TP2, 24 patients (16 HC) at TP 3 and 21 patients (17 HC) at TP4. Because of incorrect segmentation, data from 1 patient was removed after visual inspection. For two patients, a new treatment series was required within 6 months and the follow-up was rescheduled 6 months after start of the second ECT series. For HC, 11 of 19 participants were female, age ranged from 21–69 years (mean 42.26, SD = 15.69). Patient characteristics are given in Table 1. There were no significant differences between patients and HC when comparing age (*t*(48) = 0.66, *p* = 0.29, two samples *t*-test) and sex (*χ*^2^ (0, N = 52) = 1*, p =* 1 Pearson chi squared test). Healthy controls displayed no consistent changes in metabolite concentrations across referencing methods (statistical models and results are given in the Supplementary material S1). Results for patients are listed below.

**Table 1:** Demographic and clinical characteristics of 31 patients referred to ECT.

Variable	Patients
Mean age in years, min-max (SD)	45.1, 22–77 (13.8)
Mean number of ECT treatments in series, min-max (SD)	10.6, 3–18 (3.9)
Mean duration of current depressive episode in weeks, min-max (SD)	42.8, 3–150 (40.2)
Number of remitters^*^	12
Medication	
Antidepressants	12
Antipsychotics	24
Lithium	5
Benzodiazepines	0
Diagnosis for referral to ECT	
Unipolar, psychotic (F32.3/33.3)	4
Unipolar non-psychotic (F33.1/33.2)	21
Bipolar, psychotic (F31.2/31.5)	0
Bipolar, non-psychotic (F31.3/13.4)	6
Mean charge of ECT in mC, min-max (SD)	
First treatment	226.1, 76.4–404.1 (82.1)
Last treatment	252.0, 100.4–612.1 (110.16)

	Baseline	TP3	TP4
Number of participants (female)	25 (14)	24 (11)	21 (10)
Mean MADRS (SD)	34.1 (5.2)	15.6 (8.9)	14.2 (8.8)
Mean EMQ-28 (SD)	119 (35)	111 (29)	115 (37)
Number of remitters^*^	10	10	10

Upper part: patients at all timepoints, lower part: patients stratified by timepoint. *Remission: ≥ 50% reduction of baseline MADRS score + MADRS ≤ 10.

### 3.2. Clinical outcome and side effect

Patients’ MADRS scores decreased from baseline [34.1 (5.2)] to after ECT-series (TP3) [15.6 (8.9), *t*(24) = 8.94, *p* < 0.001, d = 1.79] in all but two patients and remained lower compared to baseline at 6-month follow-up in 20 out of 21 patients (TP4) [14.2 (8.8.), *t*(18) = 8.29, *p* < 0.001, d = 1.90] computed by paired samples *t*-tests. EMQ-28 scores did not differ from baseline 119 (35) to after ECT-series 111 (29) or to 6-month follow-up 115 (37).

### 3.3. Change in metabolite concentrations

For creatine, linewidth (full width at half maximum, FWHM) was in the range of 4.22–10.74 [5.72 ± 1.08 Hz (patients) 5.46 ± 0.67 (HC)] and the signal to noise ratio (SNR) was 22.84–65.53 [48 ± 7 (patients), 49 ± 6 (HC)]. The creatine ratio and water referenced metabolite concentrations for patients and controls at baseline and the percentage change to TP3 for patients are listed in Table 2. For patients, there was no significant change in creatine (tCr/H2O) over time, tested with linear mixed effects models (all *p* > 0.6).

**Table 2:** Overview of metabolite concentrations.

Group differences at baseline													
Creatine ratio (IU)							Water scaled (IU)						
	Patients	HC	*p*	%	*t*(df)	*n*		Patients	HC	*p*	%	*t*(df)	*n*
tNAA	1.41 (0.12)	1.51 (0.13)	0.007	6.8	*t*(40) = −2.85	50	tNAA	16.52 (0.94)	16.70 (0.77)	0.52	1.2	*t*(40) = −0.64	50
tCho	0.29 (0.04)	0.28 (0.03)	0.26	3.5	*t*(40) = 1.14	50	tCho	3.17 (0.47)	2.84 (0.27)	0.01	11.0	*t*(40) = 2.56	50
mI	0.69 (0.08)	0.66 (0.09)	0.36	4.4	*t*(40) = 0.93	50	mI	7.57 (1.09)	6.85 (0.91)	0.03	10.0	*t*(40) = 2.25	50
Glx	1.29 (0.16)	1.24 (0.18)	0.35	4.0	*t*(40) = 0.94	50	Glx	16.18 (1.60)	14.66 (1.79)	0.006	9.7	*t*(40) = 2.88	50

Longitudinal changes in patients													
Creatine ratio (IU)							Water scaled (IU)						
	Baseline	TP3	*p*	%	*t*(df)	*n*		Baseline	TP3	*p*	%	*t*(df)	*n*
tNAA	1.41 (0.12)	1.33 (0.11)	0.007	−5.8	*t*(60) = −2.79	31	tNAA	16.52 (0.94)	16.06 (1.10)	0.02	−2.7	*t*(60) = −2.5	31
tCho	0.29 (0.04)	0.29 (0.03)	0.36	0	*t*(40) = −0.93	31	tCho	3.17 (0.47)	3.17 (0.36)	0.54	−0.1	*t*(60) = 0.62	31
mI	0.69 (0.08)	0.68 (0.08)	0.71	1.5	*t*(60) = −0.38	31	mI	7.57 (1.09)	7.71 (1.25)	0.97	1.9	*t*(60) = 0.04	31
Glx	1.29 (0.16)	1.22 (0.19)	0.19	−5.6	*t*(60) = −1.32	31	Glx	16.18 (1.60)	15.75 (2.65)	0.42	−2.5	*t*(60) = −0.82	31

TP 3 to 4 in patients													
Creatine ratio (IU)							Water scaled (IU)						
	TP3	TP4	*p*	%	*t*(df)	*n*		TP3	TP4	*p*	%	*t*(df)	*n*
tNAA	1.33 (0.11)	1.41 (0.13)	<0.001	5.8	*t*(60) = −4.25	31	tNAA	16.06 (1.60)	17.16 (1.07)	<0.001	6.9	*t*(60) = −4.66	31

P, patients; HC, healthy controls; *p*, value of *p*; %, percentage change or difference between groups; tNAA, total N-acetylaspartate; tCho, total choline; mI, myo-Inositol; Glx, glutamate + glutamine.

#### 3.3.1. tNAA

**Figure 3:**
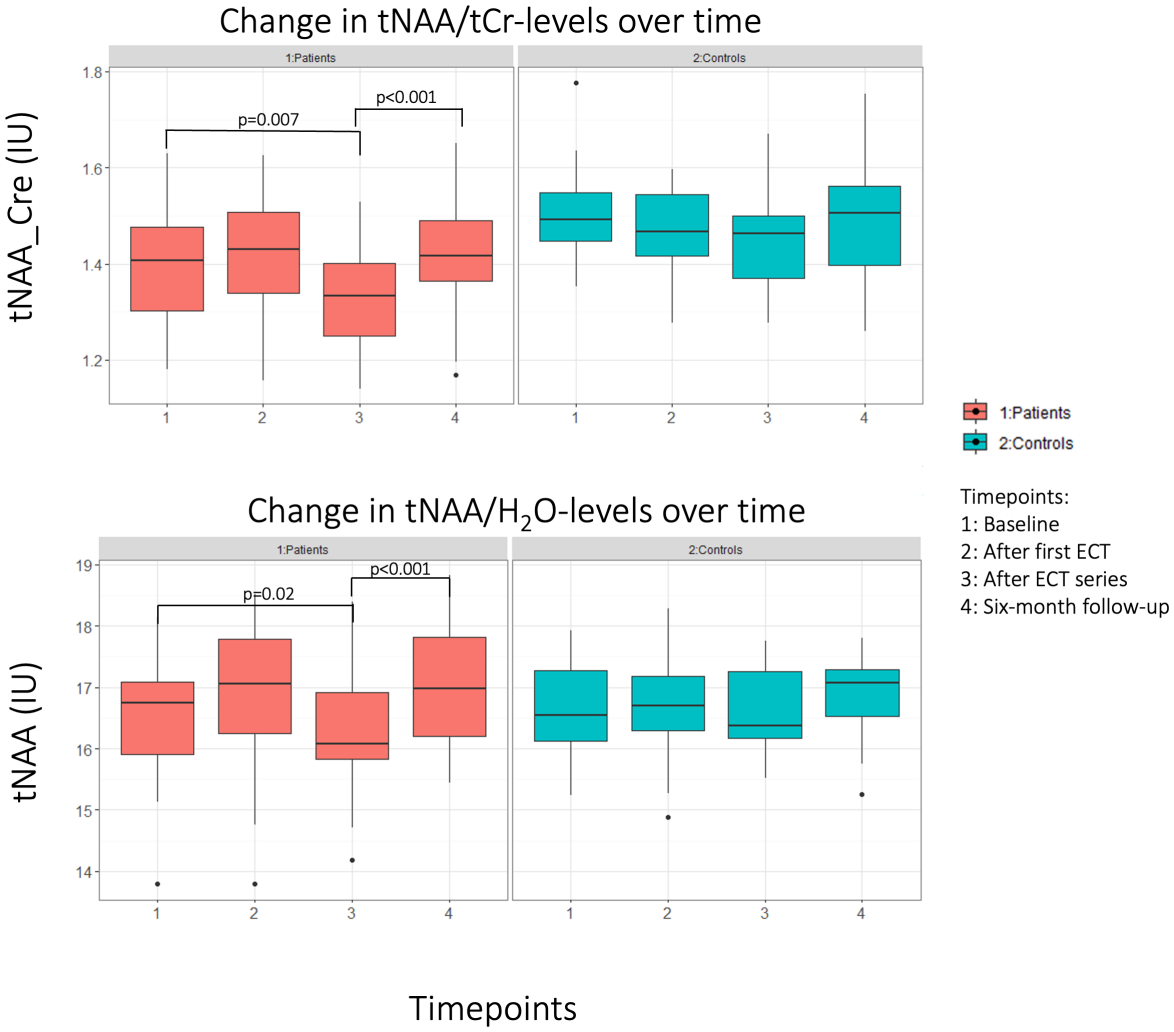
Boxplot of change in tNAA levels for patients (red) and HC (blue). Top panel: creatine ratio of tNAA. Lower panel: Water referenced tNAA values.

tNAA/tCr levels at baseline differed between patients 1.41 IU (0.12) and HC 1.51 IU (0.11) computed by a two samples *t*-test *t*(40) = −2.85, *p* = 0.007, d = −0.90. Likewise, tNAA/H_2_O levels were lower in patients 16.52 IU (0.94) compared to HC 16.70 IU (0.78), but this was not significant: *t*(40) = −0.64, *p* = 0.52. Longitudinal changes in patients were investigated with a mixed effects model, where timepoint affected both tNAA/tCr levels [*t*(60) = −2.79, *p* = 0.007] and tNAA/H_2_O levels [*t*(60) = −2.50, *p* = 0.02] in patients at TP3, resulting in a decrease compared to baseline levels. There were no significant changes in tNAA/H_2_O or tNAA/tCr from baseline to any other timepoint. When comparing TP3 to TP4 in the same model tNAA/tCr-levels increased [*t*(60) = −4.25 *p* < 0.001], for tNAA/H_2_O: [*t*(60) = −4.66, *p* < 0.001]. See Figure 3 for a visualization of longitudinal tNAA levels in patients and HC.

Linear models for tNAA/tCr and tNAA/H_2_O levels predicting MADRS or EMQ-28 were not significant for TP1, TP3 or the change between the two. tNAA/tCr and tNAA/H_2_O levels at TP3 did not differ between patients who had the voxel on the left versus on the right side compared by a two samples *t*-test.

#### 3.3.2. tCho

tCho/H_2_O was significantly lower in controls [2.84 IU (0.27)] compared to patients [3.17 IU (0.47)], *t*(40) = 2.56, *p* = 0.01, but this was not seen for tCho/Cre. There were no significant changes in tCho/tCr or tCho/H_2_O at any timepoint when compared to baseline, investigated with linear mixed effects models. There were no significant changes in tCho/H_2_O or tCho/tCr from baseline to any other timepoint, tested with a linear mixed effects model. Tested with a linear model there was no association between tCho/tCr or tCho/H_2_O and EMQ-28 at baseline or when comparing the change from before to after treatment.

#### 3.3.3. mI

Both mI/tCr and mI/H_2_O levels were higher in patients [7.57 IU (1.09), water referenced] compared to controls [6.85 IU (0.91), water referenced] at baseline, but findings were only significant for mI/H_2_O [*t*(40) = 2.25, *p* = 0.03]. There were no significant changes in mI/H_2_O or mI/tCr from baseline to any other timepoint, tested with a linear mixed effects model. Linear models for mI/tCr and mI/H_2_O levels predicting MADRS were not significant for TP1, TP3 or the change between the two.

#### 3.3.4. Glx

Patients had higher baseline Glx/H_2_O levels [16.18 IU (1.60)] than controls [14.66 IU (11.79)], *t*(40) = 2.88, *p* = 0.006. The same trend was found for Glx/tCr levels: patients [1.29 IU (0.16)], controls [1.24 IU (0.18)], however this was not significant using a two samples *t*-test [*t*(40) = 0.94, *p* = 0.35]. A linear mixed effects model showed no impact of timepoint on Glx/tCr or Glx/H_2_O.

## 4. Discussion

In this ^1^H-MRS study, we investigated creatine ratios and water referenced estimates of tNAA, tCho, mI, and Glx in 31 patients receiving ECT and 19 healthy controls. Patients were scanned at baseline, after the first ECT, after the ECT treatment series, and at six-month follow-up. HC, not receiving ECT, were scanned at similar time points. Our findings showed that ECT causes a reversible decrease in tNAA. Though the direction of the change in metabolite concentration mainly was the same across referencing methods (either increase, decrease, or no change) neither tCho, mI, or Glx displayed a significant change across referencing methods. No changes were found in any of the metabolites at the timepoint 2 h after the first ECT treatment. Possible explanations for this could be that: one single ECT treatment is not sufficient to induce metabolite changes on a level detectable with MRS or delay in detectable metabolite turnover. In the following paragraphs, we interpret our findings according to the DPR-hypothesis.

### 4.1. Disruption

We hypothesized that MRS correlates of disruption could be seen as a decrease in tNAA (a marker of neuronal integrity), an increase in tCho (membrane component), and an increase in Glx (mainly glutamate, excitatory neurotransmitter). We found a significant decrease in the patients’ tNAA levels from baseline to after the ECT series, in both creatine ratio and water referenced data. This finding is concordant with findings from several previous studies (4, 43–47).

Choline is present in cell membranes and has previously been reported to increase after ECT (47–49). An increase in choline during and after ECT treatment, especially if NAA decrease is also seen, is often understood as disruption of cell membranes. In our sample, the longitudinal change in choline was not consistent across quantification methods, and neither method yielded statistically significant change. We did not find a change in Glx concentration during the ECT treatment series. Contrary to the findings summarized in two reviews (11, 14) and our hypothesis, but in accordance with another study (50), we could not find any group differences when comparing patients and healthy controls at baseline. However, Glx is a pooled measure of glutamate and glutamine, two metabolites that are challenging to distinguish with a scanner strength of 3T. Hence, a subtle change in glutamate may not necessarily be reflected in our results.

### 4.2. Potentiation and rewiring

An increase in mI (glial proliferation marker) was hypothesized to coincide with the alleviation of depressive symptoms and would support potentiation (increased plasticity) and rewiring. Similarly, an increase in NAA or a return to baseline after the first decrease (disruption), could also be interpreted as either end of the disruptive effect or rewiring through axonal recovery after neuronal disturbances, as reviewed by Burtscher and Holtås where such a mechanism was suggested for epilepsy (51). Previously, an increase in mI has been reported in the ACC in patients treated with ECT (25). The increase was seen within one week after ECT and was interpreted as an increase in glial functioning. In our sample, we found no change in mI for any timepoint, hence the hypothesized change suggestive of potentiation or rewiring was not found. For tNAA and using either referencing method, we found an increase from after-treatment series to six-month follow-up, when the metabolite concentration was no longer different from baseline values. Though this finding might not directly imply potentiation and rewiring, our finding suggests that ECT-induced neuronal *disruption* is reversible over time.

### 4.3. Metabolite concentrations in relation to effect and side effects of ECT

Linear models for tNAA levels predicting MADRS were not significant, suggesting that the disruptive effects of the treatment could not alone explain the effect of the ECT on depressive symptoms. Linear models for mI levels predicting MADRS were not significant for TP1, TP3, or the change between the two. Other hypotheses, though not ECT-specific, have argued that both increased and reduced levels of mI might play a role in the alleviation of depressive symptoms. However, a sample studied by Njau et al. (25) could not associate the change in depression score with mI levels, and we could not find a correlation between mI levels and MADRS. Contrary to our hypothesis, mI levels were higher in patients than controls at baseline, but this was not seen for both referencing methods, and should therefore be interpreted with care.

We also hypothesized a correlation between subjective memory complaints and both tNAA-levels (increase in memory complaints due to reduced neuronal integrity: lower levels of tNAA) and tCho-levels (increased memory complaints due to disruption of cell membranes: higher levels of tCho). Linear models for tNAA levels predicting EMQ-28 were not significant, and we found no association between tCho and EMQ-28 at baseline or when comparing the change from before to after treatment. EMQ-28 is a measure of subjective everyday memory, and the correlation to depression severity seems uncertain, as both correlation and no correlation have been found (52, 53). Although the ecological validity for measuring the subjective experience may be higher for EMQ-28, it may be regarded as a measure of metamemory, i.e., what the patient reports that they forget, rather than actual forgetfulness.

### 4.4. Limitations

Our study is based on results from 31 patients and 19 healthy controls. A larger sample size would increase statistical power and possibly allow the quantification of more subtle changes in metabolite concentrations. A larger sample size would also allow for subgroup analysis based on patient heterogeneity (i.e., diagnoses, medication, comorbidity, duration of current episode etc.). Such analyses may explain results that are now interpreted as conflicting. Previous investigations have mainly been of smaller samples. Consortia like the global ECT-MRI research collaboration [GEMRIC, (54)] may give an opportunity for pooled data analysis with a larger sample size in the future.

It can be reasoned that psychotic or elderly patients who have the largest effect of ECT (55, 56) also may display the largest neurobiological changes during and after ECT. However, these groups only constitute a smaller fraction of the studied sample, as they are challenging to include in studies. Reasons for this may be that these patients carry a greater burden of disease and might therefore also not be motivated and able to give informed consent, and hence also have a greater rate of attrition.

During the statistical analysis, correction for multiple testing was not performed. The significance of the results must therefore be interpreted accordingly. Correction for multiple testing has some weaknesses as it can reduce statistical power and introduce type II errors. Hence, true differences may remain undiscovered. Additionally, the number of tests performed may be difficult to establish, as complex models yield several *p*-value, but are only one model. Our investigation was hypothesis-driven, and two referencing methods were used, strengthening results that are significant across referencing methods.

Due to the large voxel localized in the ACC region, a heterogeneous tissue composition including both gray and white matter are measured. Although Osprey (36) was used to derive tissue- and relaxation-corrected concentration estimates according to the Gasparovic method (38), reliable measurements of either gray or white matter only are not feasible. While depression has been suggested to be a disorder of brain networks (57), our study only investigated the ACC. Findings in ACC regions have been suggested as biomarkers of treatment response; larger baseline subgenual cingulate volume was found to predict response in ECT (58), and pretreatment ACC functional activity predicted response to antidepressant medication (59). However, the hippocampus and amygdala are structures known to display the largest volumetric changes following ECT (5). Both the amygdala and hippocampus are connected with the ACC, either directly or indirectly (60), and these structures also have larger gray matter fractions than the ACC. Accordingly, areas such as the amygdala and hippocampus may also display larger metabolite changes. Future MRS studies of these areas could therefore lead to better insight into ECT response in depression.

In previous MRS investigations of ECT, findings have been inhomogeneous. This could in part be due to differences in the choice of referencing method. Hence, we have used both water referenced results as well as the creatine ratio for the quantification of brain metabolites in our sample. Still, this approach does not reflect the whole variety of different MRS post processing pipelines that have been used in previous research. A more methodological comparison between methods is out of the scope of this article.

## 5. Conclusion

Using both creatine ratio and water reference for MRS-data quantification, our study indicates a decrease in tNAA levels after ECT. This was reversed to pre-ECT levels 6 months after ECT. This finding lends support to temporary disruption as suggested in the “disrupt, potentiate, and rewire” hypothesis. Longitudinal changes in mI, tCho, or Glx levels were not consistent across quantification method, and we did not find any correlation between tNAA or tCho and effect or side effects of ECT. For future research, MR-spectroscopy investigations with voxel placement in areas that have an even stronger implication in the setting of depression, and which are more affected by ECT, such as the hippocampus and amygdala, may further shed light on the disrupt, potentiate, and rewire hypothesis. Other methods, such as Diffusion Tensor Imaging and resting state functional MRI may be better to assess neuronal potentiation and rewiring. Furthermore, larger sample sizes and multi-site investigations are important to improve our understanding of brain metabolites and their role in the neurobiological underpinnings of ECT.

## Data availability statement

The original contributions presented in the study are included in the article/Supplementary material, further inquiries can be directed to the corresponding author.

## Ethics statement

The studies involving human participants were reviewed and approved by The Regional Committee for Medical and Health Research Ethics, REC South East, Norway (2013/1032). The patients/participants provided their written informed consent to participate in this study.

## Author contributions

VE: formal analysis, writing original draft, and writing-review and editing. LO and LE: conceptualization, supervision, methodology, and writing-review and editing. UK: conceptualization, methodology, and writing-review and editing. KO: conceptualization, supervision, and writing-review and editing. AC: software, formal analysis, and writing-review and editing. JH: conceptualization and writing-review and editing. FR: methodology and writing-review and editing. CB-J: methodology, statistical analysis, and writing-review and editing. All authors contributed to the article and approved the submitted version.

## Funding

Funding was received from the Western Norway Regional Health Authority (KO: no. 911986 and LO: no. 912238) and the University of Bergen (LO). The granting agencies had no role in the design and conduct of the study; collection, management, analysis, and interpretation of data; or preparation, review, or approval of the manuscript.

## Conflict of interest

The authors declare that the research was conducted in the absence of any commercial or financial relationships that could be construed as a potential conflict of interest.

## Publisher’s note

All claims expressed in this article are solely those of the authors and do not necessarily represent those of their affiliated organizations, or those of the publisher, the editors and the reviewers. Any product that may be evaluated in this article, or claim that may be made by its manufacturer, is not guaranteed or endorsed by the publisher.
