# Supplementary material for: Electroconvulsive therapy triggers a reversible decrease in brain N-acetylaspartate
Source: Front Psychiatry. 2023 Jun 9;14:1155689. doi: 10.3389/fpsyt.2023.1155689 (PMC10289547; doi:10.3389/fpsyt.2023.1155689)
Supplement: Supplementary file 1 [file Data_Sheet_1.pdf]

Supplementary material S1

Reporting standards for MRS **2**

Statistical models and output **4**

Overview of variables and abbreviations *4*

Linear mixed effects models *5*
Patients: 5
tNAA 5
tCho 7
mI 8
Glx 9
Healthy controls: 10
tNAA 10
tCho 11
mI 12
Glx 13

Linear models *14*
tNAA and MADRS 14
tNAA and EMQ 16
tCho 17
tCho and EMQ 17
mI 20
mI and MADRS 20

Reporting standards for MRS

Summary following minimum reporting standards in MRS generated in Osprey See Lin et al. 'Minimum Reporting Standards for in vivo Magnetic Resonance Spectroscopy (MRSinMRS): Experts' consensus recommendations. NMR in Biomedicine. 2021;e4484. doi.org/10.1002/nbm.4448

1. Hardware	
a. Field strength [T]	3 T
b. Manufacturer	GE
c. Model (software version if available)	MR750 (DV 26)
d. RF coils: nuclei (transmit/receive), number of channels, type, body part	1H, 32 channel head coil
e. Additional hardware	-

2. Acquisition	
a. Pulse sequence	PRESS
b. Volume of interest (VOI) locations	Anterior cingulate cortex (ACC), alternating between left and right for every other patient
c. Nominal VOI size [mm^3]	20 x 20 x 20 mm^3
d. Repetition time (TR), echo time (TE) [ms]	TR 1500 ms, TE 35 ms
e. Total number of averages per spectrum<br>i. Number of averaged spectra per subspectrum	128 total averages with 8 averages per subspectrum
f. Additional sequence parameters (spectral width in Hz, number of spectral points)	F1: 5000 Hz, 4096 points
g. Water suppression method	CHESS
h. Shimming method, reference peak, and threshold of acceptance of shim chosen	Automated 3D B 0 field mapping
i. Trigger or motion correction	None

3. Data analysis methods and outputs	
a. Analysis software	Osprey 2.4.0
b. Processing steps deviating from Osprey	None
c. Output measure	tCr, rawWaterScaled, CSFWaterScaled, TissCorrWaterScaled
d. Quantification references and assumptions, fitting model assumptions	Basis set list:<br>AscAsp,Cr,CrCH2,GABA,GPC,GSH,Gln,Glu,mI,<br>Lac,NAA,NAAG,PCh,PCr,PE,sI,Tau,MM09,<br>MM12,MM14,MM17,MM20,Lip09,Lip13,Lip20,tNAA,Glx,<br>tCho,tCr<br>Fitting method: Osprey basline knot spacing 0.40 ppm

4. Data quality	
a. SNR (Cr), linewidth (Cr) [Hz]	SNR: 48 +- 7, linewidth 5.72 +- 1.08 Hz (for patients n=31) SNR: 49 +- 6, linewidth 5.46 +- 0.67 Hz(for healthy controls n=19)
b. Data exclusion criteria	Visual inspection
c. Quality measures of postporcessing model fitting (Mean Relative Amplitude Residual)	2.86% (patients) 2.91% (healthy controls)
d. Mean spectrum created with OspreyOverview (patients, healthy controls)	

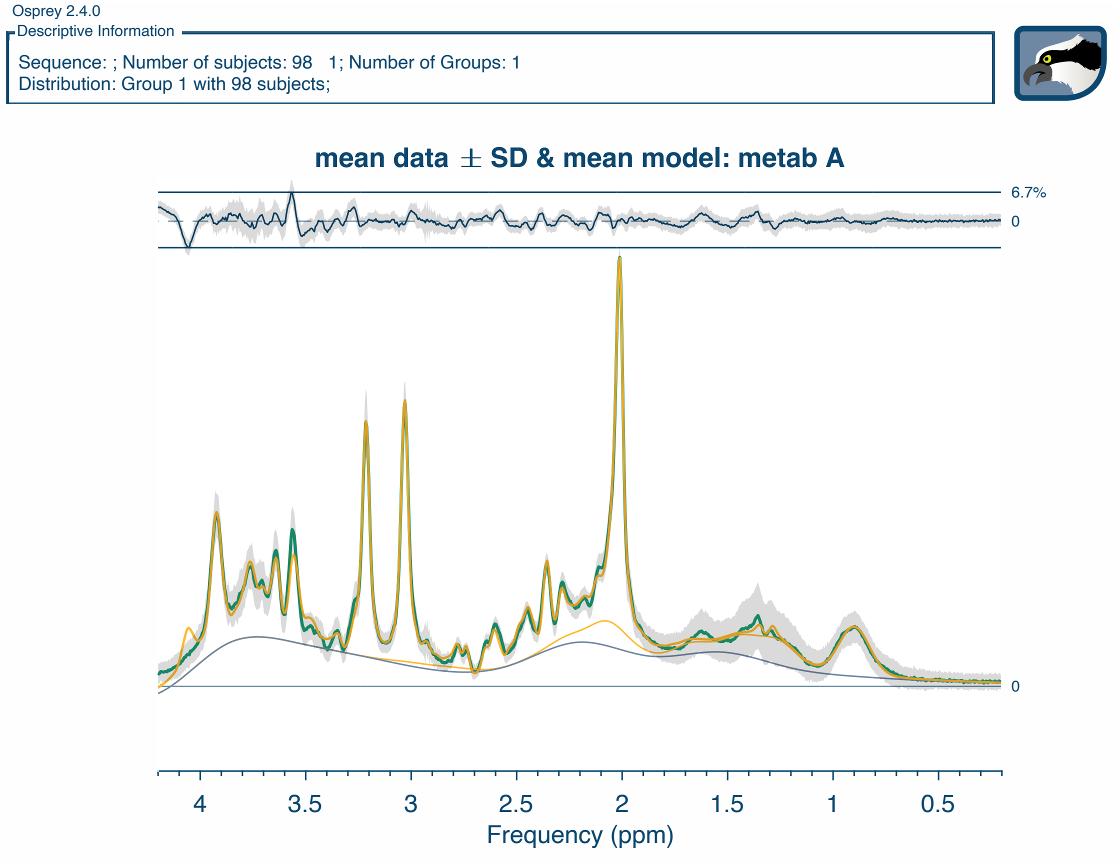

Figure 1 Mean spectrum for patients.

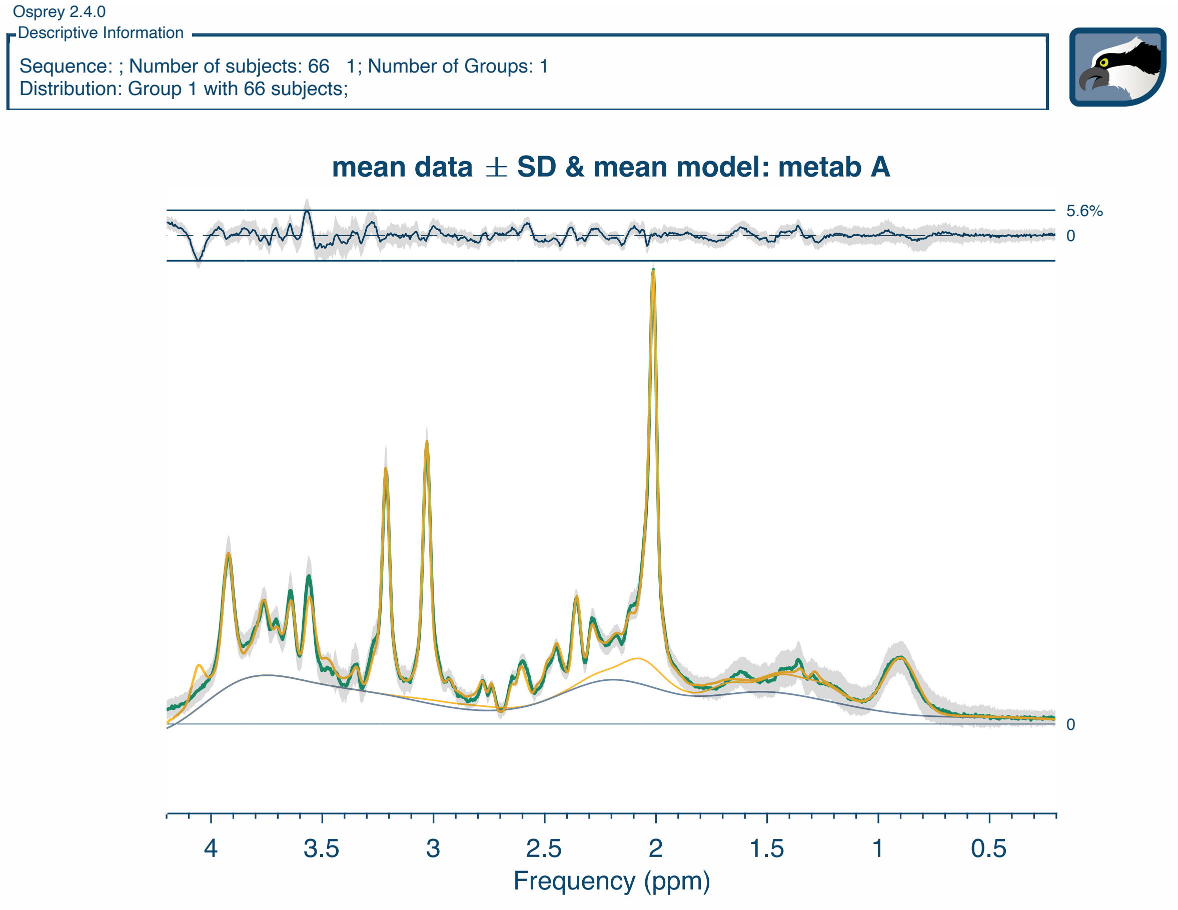

Figure 2 Mean spectrum for healthy controls.

Statistical models and output

Overview of variables and abbreviations

Variable name/abbreviation	Meaning
Kjønn	Sex
Alder_ved_start	Age at start
Antall_stoet	Number of ECTs
MADRS_0	MADRS score at baseline
MADRS_avsluttet	MADRS score at timepoint 3
EMQ_før_samlescore	EMQ score at baseline
EMQ_etter_samlescore	EMQ score at timepoint 3

Metabolites at different timepoints are indicated with *.timepoint* i.e.: tNAA.3 is tNAA-level at timepoint 3.

Linear mixed effects models

Patients:

tNAA

tNAA/tCr

```
## Linear mixed-effects model fit by REML
##   Data: lme_tNAA_long_patients
##         AIC       BIC  logLik
##   -119.7632 -95.21973 69.8816
##
## Random effects:
##  Formula: ~1 | SubjectID
##         (Intercept)   Residual
## StdDev:   0.1030499 0.06371544
##
## Fixed effects:  tNAA ~ VisitID + kjønn + alder_ved_start + antall_stoet + remission
##                      Value  Std.Error DF   t-value p-value
## (Intercept)      1.5295318 0.09718771 60 15.737914  0.0000
## VisitID2         0.0084457 0.01848309 60  0.456941  0.6494
## VisitID3        -0.0527349 0.01892504 60 -2.786512  0.0071
## VisitID4         0.0329640 0.02026721 60  1.626471  0.1091
## kjønn            0.0184664 0.04192199 26  0.440494  0.6632
## alder_ved_start -0.0034983 0.00153356 26 -2.281133  0.0310
## antall_stoet     0.0018850 0.00567179 26  0.332339  0.7423
## remissionTRUE   -0.0061522 0.04619237 26 -0.133185  0.8951
##  Correlation:
##                 (Intr) VstID2 VstID3 VstID4 kjønn  aldr__ antll_
## VisitID2        -0.088
## VisitID3        -0.088  0.487
## VisitID4        -0.077  0.444  0.473
## kjønn            0.022 -0.006 -0.008  0.035
## alder_ved_start -0.628 -0.011 -0.027 -0.046 -0.299
## antall_stoet    -0.706  0.006  0.019  0.017 -0.010  0.007
## remissionTRUE   -0.365  0.007  0.007 -0.012 -0.025 -0.131  0.448
##
## Standardized Within-Group Residuals:
##         Min          Q1         Med          Q3         Max
## -1.91346156 -0.53920658  0.04546074  0.56657442  2.12029958
##
## Number of Observations: 94
## Number of Groups: 31
```

tNAA/H2O

```
## Linear mixed-effects model fit by REML
##   Data: lme_tNAA_long_TC_patients
##        AIC      BIC    logLik
##   292.9494 317.4928 -136.4747
##
## Random effects:
##  Formula: ~1 | SubjectID
##         (Intercept)  Residual
## StdDev:   0.8772521 0.7638231
##
## Fixed effects:  tNAA ~ VisitID + kjønn + alder_ved_start + antall_stoet + remission
##                     Value Std.Error DF   t-value p-value
## (Intercept)     16.065743 0.8755396 60 18.349534  0.0000
## VisitID2         0.355018 0.2210377 60  1.606141  0.1135
## VisitID3        -0.564724 0.2254631 60 -2.504728  0.0150
## VisitID4         0.550294 0.2400208 60  2.292692  0.0254
## kjønn           -0.328790 0.3780389 26 -0.869725  0.3924
## alder_ved_start  0.016322 0.0136974 26  1.191585  0.2442
## antall_stoet    -0.012359 0.0509170 26 -0.242727  0.8101
## remissionTRUE   -0.055505 0.4175065 26 -0.132943  0.8953
##  Correlation:
##                 (Intr) VstID2 VstID3 VstID4 kjønn  aldr__ antll_
## VisitID2        -0.117
## VisitID3        -0.117  0.487
## VisitID4        -0.102  0.448  0.474
## kjønn            0.013 -0.007 -0.008  0.039
## alder_ved_start -0.616 -0.014 -0.034 -0.055 -0.302
## antall_stoet    -0.705  0.007  0.024  0.020 -0.004  0.003
## remissionTRUE   -0.374  0.010  0.009 -0.015 -0.018 -0.129  0.455
##
## Standardized Within-Group Residuals:
##         Min          Q1         Med          Q3         Max
## -1.50849971 -0.64380607 -0.08408892  0.61903170  2.39544670
##
## Number of Observations: 94
## Number of Groups: 31
```

Contrast: time point 3 vs time point 4

tNAA/H2O

```
contrast::contrast(tNAA_lme_visit_TC,list(VisitID="3", alder_ved_start=0, kjønn=0, antall_stoet=0, remission=FALS
E), list(VisitID="4", alder_ved_start=0, kjønn=0, antall_stoet=0, remission=FALSE))
```

```
## lme model parameter contrast
##
##   Contrast      S.E.     Lower      Upper     t df Pr(>|t|)
## -1.115018 0.2390229 -1.593135 -0.6369005 -4.66 60        0
```

tNAA/tCr

```
contrast::contrast(tNAA_lme_Visit,list(VisitID="3", alder_ved_start=0, kjønn=0, antall_stoet=0, remission=FALS
E), list(VisitID="4", alder_ved_start=0, kjønn=0, antall_stoet=0,remission=FALSE))
```

```
## lme model parameter contrast
##
##     Contrast       S.E.      Lower       Upper     t df Pr(>|t|)
## -0.08569888 0.02014352 -0.1259919 -0.04540584 -4.25 60    1e-04
```

tCho

tCho/tCr

```
## Linear mixed-effects model fit by REML
##   Data: lme_tCho_tCr_long_patients
##         AIC       BIC   logLik
##   -323.4104 -298.8669 171.7052
##
## Random effects:
##  Formula: ~1 | SubjectID
##         (Intercept)   Residual
## StdDev:  0.02586555 0.02083341
##
## Fixed effects:  tCho ~ VisitID + remission + kjønn + alder_ved_start + antall_stoet
##                       Value   Std.Error DF   t-value p-value
## (Intercept)      0.29822075 0.025411577 60 11.735625  0.0000
## VisitID2        -0.00626903 0.006032531 60 -1.039204  0.3029
## VisitID3        -0.00570825 0.006159219 60 -0.926782  0.3578
## VisitID4        -0.00507517 0.006566538 60 -0.772883  0.4426
## remissionTRUE   -0.00828547 0.012108907 26 -0.684246  0.4999
## kjønn            0.02445651 0.010971374 26  2.229120  0.0347
## alder_ved_start  0.00014677 0.000398490 26  0.368306  0.7156
## antall_stoet    -0.00186237 0.001479229 26 -1.259017  0.2192
##  Correlation:
##                 (Intr) VstID2 VstID3 VstID4 rmTRUE kjønn  aldr__
## VisitID2        -0.110
## VisitID3        -0.110  0.487
## VisitID4        -0.096  0.447  0.474
## remissionTRUE   -0.372  0.009  0.009 -0.015
## kjønn            0.015 -0.007 -0.008  0.038 -0.020
## alder_ved_start -0.619 -0.014 -0.032 -0.053 -0.129 -0.301
## antall_stoet    -0.705  0.007  0.023  0.019  0.453 -0.005  0.004
##
## Standardized Within-Group Residuals:
##        Min         Q1        Med         Q3        Max
## -1.9346436 -0.5119534 -0.0296000  0.5068872  3.0953935
##
## Number of Observations: 94
## Number of Groups: 31
```

tNAA/tH2O

```
## Linear mixed-effects model fit by REML
##   Data: lme_tCho_long_TC_patients
##        AIC      BIC    logLik
##   90.50502 115.0485 -35.25251
##
## Random effects:
##  Formula: ~1 | SubjectID
##         (Intercept)  Residual
## StdDev:   0.2460443 0.2419211
##
## Fixed effects:  tCho ~ VisitID + remission + kjønn + alder_ved_start + antall_stoet
##                      Value  Std.Error DF   t-value p-value
## (Intercept)      2.8699436 0.25269468 60 11.357357  0.0000
## VisitID2        -0.0242124 0.06993755 60 -0.346200  0.7304
## VisitID3        -0.0443175 0.07122489 60 -0.622219  0.5362
## VisitID4        -0.0184275 0.07564384 60 -0.243609  0.8084
## remissionTRUE   -0.0721639 0.12062161 26 -0.598267  0.5548
## kjønn            0.1564757 0.10909059 26  1.434365  0.1634
## alder_ved_start  0.0122376 0.00393675 26  3.108548  0.0045
## antall_stoet    -0.0274879 0.01467035 26 -1.873705  0.0723
##  Correlation:
##                 (Intr) VstID2 VstID3 VstID4 rmTRUE kjønn  aldr__
## VisitID2        -0.129
## VisitID3        -0.129  0.487
## VisitID4        -0.112  0.450  0.474
## remissionTRUE   -0.378  0.011  0.010 -0.017
## kjønn            0.008 -0.008 -0.007  0.039 -0.015
## alder_ved_start -0.610 -0.015 -0.036 -0.057 -0.128 -0.303
## antall_stoet    -0.705  0.008  0.026  0.020  0.458 -0.001  0.002
##
## Standardized Within-Group Residuals:
##        Min         Q1        Med         Q3        Max
## -2.0376915 -0.5602323 -0.1009668  0.4388930  2.4145753
##
## Number of Observations: 94
## Number of Groups: 31
```

mI

mI/tCr

```
## Linear mixed-effects model fit by REML
##   Data: lme_mI_tCr_long_patients
##         AIC       BIC  logLik
##   -115.6372 -91.09372 67.8186
##
## Random effects:
##  Formula: ~1 | SubjectID
##         (Intercept)   Residual
## StdDev:   0.0658027 0.07532644
##
## Fixed effects:  mI ~ VisitID + kjønn + alder_ved_start + antall_stoet + remission
##                      Value  Std.Error DF   t-value p-value
## (Intercept)      0.6654363 0.07059280 60  9.426405  0.0000
## VisitID2        -0.0353092 0.02174765 60 -1.623589  0.1097
## VisitID3        -0.0084086 0.02210201 60 -0.380445  0.7050
## VisitID4        -0.0071867 0.02340213 60 -0.307098  0.7598
## kjønn           -0.0270313 0.03045218 26 -0.887664  0.3829
## alder_ved_start  0.0005988 0.00109304 26  0.547833  0.5885
## antall_stoet     0.0011457 0.00408801 26  0.280253  0.7815
## remissionTRUE    0.0039765 0.03372937 26  0.117895  0.9071
##  Correlation:
##                 (Intr) VstID2 VstID3 VstID4 kjønn  aldr__ antll_
## VisitID2        -0.144
## VisitID3        -0.144  0.487
## VisitID4        -0.125  0.452  0.475
## kjønn            0.003 -0.008 -0.007  0.039
## alder_ved_start -0.603 -0.016 -0.038 -0.060 -0.304
## antall_stoet    -0.705  0.008  0.028  0.021  0.003  0.000
## remissionTRUE   -0.384  0.013  0.010 -0.019 -0.010 -0.127  0.463
##
## Standardized Within-Group Residuals:
##          Min           Q1          Med           Q3          Max
## -2.524036322 -0.445872418  0.001972052  0.561647882  2.268864538
##
## Number of Observations: 94
## Number of Groups: 31
```

mI/H2O

```
## Linear mixed-effects model fit by REML
##   Data: lme_Ins_long_TC_patients
##        AIC      BIC    logLik
##   304.2092 328.7527 -142.1046
##
## Random effects:
##  Formula: ~1 | SubjectID
##         (Intercept)  Residual
## StdDev:   0.7581557 0.8643571
##
## Fixed effects:  Ins ~ VisitID + remission + kjønn + alder_ved_start + antall_stoet
##                     Value Std.Error DF   t-value p-value
## (Intercept)      6.381226 0.8122974 60  7.855775  0.0000
## VisitID2        -0.302523 0.2495592 60 -1.212228  0.2302
## VisitID3         0.010374 0.2536399 60  0.040902  0.9675
## VisitID4         0.010036 0.2685817 60  0.037368  0.9703
## remissionTRUE    0.104140 0.3881094 26  0.268325  0.7906
## kjønn           -0.492934 0.3504176 26 -1.406704  0.1714
## alder_ved_start  0.031992 0.0125797 26  2.543112  0.0173
## antall_stoet     0.001607 0.0470434 26  0.034163  0.9730
##  Correlation:
##                 (Intr) VstID2 VstID3 VstID4 rmTRUE kjønn  aldr__
## VisitID2        -0.144
## VisitID3        -0.143  0.487
## VisitID4        -0.124  0.452  0.475
## remissionTRUE   -0.383  0.013  0.010 -0.019
## kjønn            0.003 -0.008 -0.007  0.039 -0.010
## alder_ved_start -0.603 -0.016 -0.038 -0.060 -0.127 -0.304
## antall_stoet    -0.705  0.008  0.028  0.021  0.463  0.003  0.000
##
## Standardized Within-Group Residuals:
##         Min          Q1         Med          Q3         Max
## -2.61532051 -0.53872927 -0.05526131  0.43270349  2.11073543
##
## Number of Observations: 94
## Number of Groups: 31
```

Glx

Glx/tCr

```
## Linear mixed-effects model fit by REML
##   Data: lme_Glx_tCr_long_patients
##         AIC      BIC   logLik
##   0.2802517 24.82372 9.859874
##
## Random effects:
##  Formula: ~1 | SubjectID
##         (Intercept) Residual
## StdDev:   0.1007679 0.155992
##
## Fixed effects:  Glx ~ VisitID + remission + kjønn + alder_ved_start + antall_stoet
##                      Value  Std.Error DF   t-value p-value
## (Intercept)      1.3202518 0.12141163 60 10.874179  0.0000
## VisitID2        -0.0277468 0.04491923 60 -0.617703  0.5391
## VisitID3        -0.0598748 0.04546370 60 -1.316980  0.1929
## VisitID4        -0.0507866 0.04786017 60 -1.061146  0.2929
## remissionTRUE   -0.0625731 0.05803499 26 -1.078197  0.2908
## kjønn            0.0895633 0.05218570 26  1.716242  0.0980
## alder_ved_start -0.0016951 0.00185252 26 -0.915047  0.3686
## antall_stoet     0.0019115 0.00698792 26  0.273548  0.7866
##  Correlation:
##                 (Intr) VstID2 VstID3 VstID4 rmTRUE kjønn  aldr__
## VisitID2        -0.174
## VisitID3        -0.174  0.488
## VisitID4        -0.150  0.457  0.475
## remissionTRUE   -0.394  0.016  0.011 -0.022
## kjønn           -0.010 -0.008 -0.005  0.037  0.001
## alder_ved_start -0.588 -0.018 -0.043 -0.064 -0.125 -0.307
## antall_stoet    -0.704  0.008  0.032  0.021  0.473  0.012 -0.004
##
## Standardized Within-Group Residuals:
##        Min         Q1        Med         Q3        Max
## -2.9008295 -0.5063321  0.1071617  0.5023713  2.7119901
##
## Number of Observations: 94
## Number of Groups: 31
```

Glx/H2O

```
## Linear mixed-effects model fit by REML
##   Data: lme_Glx_long_patients
##        AIC     BIC    logLik
##   438.5495 463.093 -209.2748
##
## Random effects:
##  Formula: ~1 | SubjectID
##         (Intercept) Residual
## StdDev:    0.745952 2.141612
##
## Fixed effects:  Glx ~ VisitID + remission + kjønn + alder_ved_start + antall_stoet
##                      Value Std.Error DF   t-value p-value
## (Intercept)      15.013890 1.3023398 60 11.528397  0.0000
## VisitID2        -0.092720 0.6141497 60 -0.150972  0.8805
## VisitID3        -0.505671 0.6176474 60 -0.818705  0.4162
## VisitID4        -0.406041 0.6446180 60 -0.629894  0.5312
## remissionTRUE   -0.782356 0.6194701 26 -1.262944  0.2178
## kjønn            0.565843 0.5528359 26  1.023527  0.3155
## alder_ved_start  0.031164 0.0192501 26  1.618891  0.1175
## antall_stoet    -0.019126 0.0739117 26 -0.258770  0.7979
##  Correlation:
##                 (Intr) VstID2 VstID3 VstID4 rmTRUE kjønn  aldr__
## VisitID2        -0.224
## VisitID3        -0.224  0.489
## VisitID4        -0.193  0.465  0.475
## remissionTRUE   -0.412  0.020  0.013 -0.027
## kjønn           -0.034 -0.008 -0.001  0.028  0.025
## alder_ved_start -0.561 -0.020 -0.046 -0.065 -0.123 -0.309
## antall_stoet    -0.701  0.008  0.035  0.019  0.490  0.027 -0.007
##
## Standardized Within-Group Residuals:
##        Min         Q1        Med         Q3        Max
## -3.1266967 -0.6127670  0.1114451  0.5486321  2.3119212
##
## Number of Observations: 94
## Number of Groups: 31
```

Healthy controls:

tNAA

tNAA/tCr

```
## Linear mixed-effects model fit by REML
##   Data: lme_tNAA_long_controls
##         AIC       BIC   logLik
##   -73.17406 -56.41931 44.58703
##
## Random effects:
##  Formula: ~1 | SubjectID
##         (Intercept)   Residual
## StdDev:  0.02567718 0.09156845
##
## Fixed effects:  tNAA ~ VisitID + kjønn + alder_ved_start
##                      Value  Std.Error DF  t-value p-value
## (Intercept)      1.6635030 0.04085657 44 40.71568  0.0000
## VisitID2        -0.0398331 0.03193572 44 -1.24729  0.2189
## VisitID3        -0.0540036 0.03193406 44 -1.69110  0.0979
## VisitID4        -0.0135906 0.03158069 44 -0.43035  0.6690
## kjønn            0.0108488 0.02652298 16  0.40903  0.6879
## alder_ved_start -0.0037655 0.00082534 16 -4.56230  0.0003
##  Correlation:
##                 (Intr) VstID2 VstID3 VstID4 kjønn
## VisitID2        -0.359
## VisitID3        -0.366  0.484
## VisitID4        -0.349  0.490  0.492
## kjønn           -0.082 -0.012 -0.015 -0.033
## alder_ved_start -0.783 -0.019 -0.009 -0.032 -0.220
##
## Standardized Within-Group Residuals:
##        Min         Q1        Med         Q3        Max
## -2.1908499 -0.5994806  0.0038574  0.5205497  2.2003362
##
## Number of Observations: 66
## Number of Groups: 19
```

tNAA/H2O

```
## Linear mixed-effects model fit by REML
##   Data: lme_tNAA_long_TC_controls
##        AIC      BIC    logLik
##    169.682 186.4368 -76.84101
##
## Random effects:
##  Formula: ~1 | SubjectID
##         (Intercept)  Residual
## StdDev:   0.5773001 0.5930886
##
## Fixed effects:  tNAA ~ VisitID + kjønn + alder_ved_start
##                     Value Std.Error DF  t-value p-value
## (Intercept)     16.275434 0.4586215 44 35.48772  0.0000
## VisitID2         0.028281 0.2073780 44  0.13638  0.8921
## VisitID3        -0.075768 0.2076274 44 -0.36492  0.7169
## VisitID4         0.267730 0.2085246 44  1.28392  0.2059
## kjønn            0.358977 0.3189865 16  1.12537  0.2770
## alder_ved_start  0.006471 0.0101076 16  0.64017  0.5311
##  Correlation:
##                 (Intr) VstID2 VstID3 VstID4 kjønn
## VisitID2        -0.205
## VisitID3        -0.208  0.480
## VisitID4        -0.197  0.478  0.488
## kjønn           -0.074 -0.007 -0.011 -0.024
## alder_ved_start -0.857 -0.011 -0.007 -0.027 -0.232
##
## Standardized Within-Group Residuals:
##          Min           Q1          Med           Q3          Max
## -2.205193755 -0.523942085  0.009908716  0.625343853  2.186753185
##
## Number of Observations: 66
## Number of Groups: 19
```

tCho

tCho/H2O

```
## Linear mixed-effects model fit by REML
##   Data: lme_tCho_long_TC_controls
##        AIC     BIC    logLik
##   28.13245 44.8872 -6.066223
##
## Random effects:
##  Formula: ~1 | SubjectID
##         (Intercept)  Residual
## StdDev:   0.2049205 0.1756513
##
## Fixed effects:  tCho ~ VisitID + kjønn + alder_ved_start
##                     Value  Std.Error DF   t-value p-value
## (Intercept)     2.5446242 0.15587092 44 16.325201  0.0000
## VisitID2        0.0507015 0.06143599 44  0.825274  0.4137
## VisitID3        0.0573694 0.06152588 44  0.932443  0.3562
## VisitID4        0.1388000 0.06198846 44  2.239126  0.0303
## kjønn           0.2589024 0.10901670 16  2.374888  0.0304
## alder_ved_start 0.0045237 0.00346705 16  1.304754  0.2104
##  Correlation:
##                 (Intr) VstID2 VstID3 VstID4 kjønn
## VisitID2        -0.179
## VisitID3        -0.181  0.479
## VisitID4        -0.171  0.476  0.487
## kjønn           -0.073 -0.006 -0.010 -0.021
## alder_ved_start -0.866 -0.010 -0.006 -0.026 -0.234
##
## Standardized Within-Group Residuals:
##        Min         Q1        Med         Q3        Max
## -2.9907714 -0.4986833  0.1201067  0.5784538  1.4537192
##
## Number of Observations: 66
## Number of Groups: 19
```

tCho/tCr

```
## Linear mixed-effects model fit by REML
##   Data: lme_tCho_tCr_long_controls
##         AIC       BIC   logLik
##   -227.3958 -210.6411 121.6979
##
## Random effects:
##  Formula: ~1 | SubjectID
##         (Intercept)   Residual
## StdDev:  0.02597543 0.02052001
##
## Fixed effects:  tCho ~ VisitID + kjønn + alder_ved_start
##                       Value   Std.Error DF   t-value p-value
## (Intercept)      0.28673551 0.019445668 44 14.745470  0.0000
## VisitID2        -0.00231580 0.007177925 44 -0.322628  0.7485
## VisitID3        -0.00325324 0.007189208 44 -0.452517  0.6531
## VisitID4         0.00528542 0.007253247 44  0.728697  0.4700
## kjønn            0.01994272 0.013626522 16  1.463523  0.1627
## alder_ved_start -0.00032713 0.000434048 16 -0.753663  0.4620
##  Correlation:
##                 (Intr) VstID2 VstID3 VstID4 kjønn
## VisitID2        -0.167
## VisitID3        -0.169  0.479
## VisitID4        -0.160  0.475  0.486
## kjønn           -0.073 -0.005 -0.010 -0.020
## alder_ved_start -0.869 -0.009 -0.006 -0.025 -0.234
##
## Standardized Within-Group Residuals:
##        Min         Q1        Med         Q3        Max
## -3.3028769 -0.5491546  0.1191768  0.5051243  1.4614491
##
## Number of Observations: 66
## Number of Groups: 19
```

mI

mI/H2O

```
## Linear mixed-effects model fit by REML
##   Data: lme_Ins_long_TC_controls
##        AIC      BIC    logLik
##   167.4409 184.1956 -75.72044
##
## Random effects:
##  Formula: ~1 | SubjectID
##         (Intercept)  Residual
## StdDev:   0.6627239 0.5588633
##
## Fixed effects:  Ins ~ VisitID + kjønn + alder_ved_start
##                    Value Std.Error DF   t-value p-value
## (Intercept)     6.155558 0.5024059 44 12.252160  0.0000
## VisitID2        0.124702 0.1954733 44  0.637950  0.5268
## VisitID3        0.344828 0.1957637 44  1.761448  0.0851
## VisitID4        0.630902 0.1972907 44  3.197829  0.0026
## kjønn           0.811281 0.3515291 16  2.307863  0.0347
## alder_ved_start 0.009181 0.0111832 16  0.820928  0.4238
##  Correlation:
##                 (Intr) VstID2 VstID3 VstID4 kjønn
## VisitID2        -0.176
## VisitID3        -0.178  0.479
## VisitID4        -0.169  0.475  0.487
## kjønn           -0.073 -0.006 -0.010 -0.021
## alder_ved_start -0.867 -0.010 -0.006 -0.025 -0.234
##
## Standardized Within-Group Residuals:
##         Min          Q1         Med          Q3         Max
## -1.76950421 -0.51161471 -0.04166127  0.47891944  2.50107636
##
## Number of Observations: 66
## Number of Groups: 19
```

mI/tCr

```
## Linear mixed-effects model fit by REML
##   Data: lme_mI_tCr_long_controls
##         AIC       BIC   logLik
##   -113.0941 -96.33933 64.54704
##
## Random effects:
##  Formula: ~1 | SubjectID
##         (Intercept)   Residual
## StdDev:  0.07438286 0.05168198
##
## Fixed effects:  mI ~ VisitID + kjønn + alder_ved_start
##                      Value  Std.Error DF   t-value p-value
## (Intercept)      0.6784015 0.05452041 44 12.443074  0.0000
## VisitID2        -0.0054823 0.01808125 44 -0.303202  0.7632
## VisitID3         0.0115450 0.01811250 44  0.637407  0.5272
## VisitID4         0.0382608 0.01831130 44  2.089462  0.0425
## kjønn            0.0655145 0.03829683 16  1.710703  0.1065
## alder_ved_start -0.0008761 0.00122272 16 -0.716479  0.4840
##  Correlation:
##                 (Intr) VstID2 VstID3 VstID4 kjønn
## VisitID2        -0.150
## VisitID3        -0.152  0.479
## VisitID4        -0.143  0.473  0.486
## kjønn           -0.072 -0.005 -0.009 -0.018
## alder_ved_start -0.874 -0.008 -0.006 -0.023 -0.235
##
## Standardized Within-Group Residuals:
##         Min          Q1         Med          Q3         Max
## -1.73701480 -0.47042023  0.03071608  0.49441996  2.10598609
##
## Number of Observations: 66
## Number of Groups: 19
```

Glx

Glx/tCr

```
## Linear mixed-effects model fit by REML
##   Data: lme_glx_long_tCr_controls
##         AIC       BIC   logLik
##   -30.39326 -13.63851 23.19663
##
## Random effects:
##  Formula: ~1 | SubjectID
##         (Intercept)  Residual
## StdDev:  0.09139411 0.1165484
##
## Fixed effects:  Glx ~ VisitID + kjønn + alder_ved_start
##                      Value  Std.Error DF   t-value p-value
## (Intercept)      1.4640670 0.07779121 44 18.820468  0.0000
## VisitID2         0.0351311 0.04073475 44  0.862436  0.3931
## VisitID3         0.0296709 0.04077050 44  0.727755  0.4706
## VisitID4         0.0587146 0.04079179 44  1.439372  0.1571
## kjønn            0.0193376 0.05361208 16  0.360695  0.7230
## alder_ved_start -0.0055221 0.00169130 16 -3.264997  0.0049
##  Correlation:
##                 (Intr) VstID2 VstID3 VstID4 kjønn
## VisitID2        -0.238
## VisitID3        -0.242  0.481
## VisitID4        -0.229  0.481  0.489
## kjønn           -0.076 -0.008 -0.012 -0.026
## alder_ved_start -0.844 -0.013 -0.008 -0.029 -0.231
##
## Standardized Within-Group Residuals:
##         Min          Q1         Med          Q3         Max
## -2.20286646 -0.41972598 -0.09016612  0.57303136  2.13797283
##
## Number of Observations: 66
## Number of Groups: 19
```

Glx/H2O

```
## Linear mixed-effects model fit by REML
##   Data: lme_glx_long_TC_controls
##        AIC      BIC    logLik
##   273.8027 290.5574 -128.9013
##
## Random effects:
##  Formula: ~1 | SubjectID
##         (Intercept) Residual
## StdDev:   0.8982463 1.540197
##
## Fixed effects:  Glx ~ VisitID + kjønn + alder_ved_start
##                     Value Std.Error DF   t-value p-value
## (Intercept)     15.497552 0.8704330 44 17.804416  0.0000
## VisitID2         0.810918 0.5379537 44  1.507412  0.1389
## VisitID3         0.861332 0.5382073 44  1.600373  0.1167
## VisitID4         1.167813 0.5359863 44  2.178811  0.0347
## kjønn            0.423728 0.5899903 16  0.718195  0.4830
## alder_ved_start -0.024075 0.0185108 16 -1.300584  0.2118
##  Correlation:
##                 (Intr) VstID2 VstID3 VstID4 kjønn
## VisitID2        -0.282
## VisitID3        -0.287  0.482
## VisitID4        -0.273  0.484  0.490
## kjønn           -0.078 -0.009 -0.014 -0.029
## alder_ved_start -0.825 -0.015 -0.008 -0.031 -0.227
##
## Standardized Within-Group Residuals:
##         Min          Q1         Med          Q3         Max
## -2.00232178 -0.60594361 -0.02985292  0.50975524  2.34145909
##
## Number of Observations: 66
## Number of Groups: 19
```

Linear models

tNAA and MADRS

tNAA/H2O

```
##
## Call:
## lm(formula = MADRS_0 ~ tNAA.1 + kjønn + alder_ved_start, data = MADRS_data.1,
##     na.action = na.omit)
##
## Residuals:
##     Min      1Q  Median      3Q     Max
## -7.6580 -3.7759 -0.5163  4.6070  9.9553
##
## Coefficients:
##                 Estimate Std. Error t value Pr(>|t|)
## (Intercept)     33.72870   19.10320   1.766    0.092 .
## tNAA.1           0.20877    1.17796   0.177    0.861
## kjønn           -1.69814    2.31343  -0.734    0.471
## alder_ved_start -0.05096    0.07883  -0.646    0.525
## ---
## Signif. codes:  0 '***' 0.001 '**' 0.01 '*' 0.05 '.' 0.1 ' ' 1
##
## Residual standard error: 5.218 on 21 degrees of freedom
##   (6 observations deleted due to missingness)
## Multiple R-squared:  0.07321,    Adjusted R-squared:  -0.05918
## F-statistic: 0.553 on 3 and 21 DF,  p-value: 0.6518
```

```
##
## Call:
## lm(formula = MADRS_avsluttet ~ tNAA.3 + kjønn + alder_ved_start,
##     data = MADRS_data.1, na.action = na.omit)
##
## Residuals:
##     Min      1Q  Median      3Q     Max
## -14.839  -4.555  -2.297   6.421  15.184
##
## Coefficients:
##                 Estimate Std. Error t value Pr(>|t|)
## (Intercept)     73.34366   24.26115   3.023  0.00672 **
## tNAA.3          -3.35288    1.55974  -2.150  0.04400 *
## kjønn           -1.67042    3.37409  -0.495  0.62594
## alder_ved_start -0.09392    0.12130  -0.774  0.44782
## ---
## Signif. codes:  0 '***' 0.001 '**' 0.01 '*' 0.05 '.' 0.1 ' ' 1
##
## Residual standard error: 7.884 on 20 degrees of freedom
##   (7 observations deleted due to missingness)
## Multiple R-squared:  0.2637, Adjusted R-squared:  0.1532
## F-statistic: 2.388 on 3 and 20 DF,  p-value: 0.09926
```

```
##
## Call:
## lm(formula = delta_MADRS ~ delta_NAA + kjønn + alder_ved_start,
##     data = MADRS_data.1, na.action = na.omit)
##
## Residuals:
##      Min       1Q   Median       3Q      Max
## -18.6065  -4.8896  -0.9657   8.6347  19.9456
##
## Coefficients:
##                  Estimate Std. Error t value Pr(>|t|)
## (Intercept)     -18.34619    7.67284  -2.391   0.0286 *
## delta_NAA         3.10225    2.30484   1.346   0.1960
## kjønn            -1.15965    4.98550  -0.233   0.8188
## alder_ved_start  -0.06204    0.16177  -0.384   0.7061
## ---
## Signif. codes:  0 '***' 0.001 '**' 0.01 '*' 0.05 '.' 0.1 ' ' 1
##
## Residual standard error: 10.73 on 17 degrees of freedom
##   (10 observations deleted due to missingness)
## Multiple R-squared:  0.1188, Adjusted R-squared:  -0.03666
## F-statistic: 0.7642 on 3 and 17 DF,  p-value: 0.5296
```

tNAA/tCr

```
##
## Call:
## lm(formula = MADRS_0 ~ tNAA.1 + kjønn + alder_ved_start, data = MADRS_data.1,
##     na.action = na.omit)
##
## Residuals:
##     Min      1Q  Median      3Q     Max
## -7.4623 -3.6134 -0.7475  3.8229 11.2521
##
## Coefficients:
##                 Estimate Std. Error t value Pr(>|t|)
## (Intercept)     51.90861   15.82719   3.280  0.00358 **
## tNAA.1          -9.46062    9.87640  -0.958  0.34901
## kjønn           -1.53849    2.23420  -0.689  0.49861
## alder_ved_start -0.08456    0.08417  -1.005  0.32651
## ---
## Signif. codes:  0 '***' 0.001 '**' 0.01 '*' 0.05 '.' 0.1 ' ' 1
##
## Residual standard error: 5.111 on 21 degrees of freedom
##   (6 observations deleted due to missingness)
## Multiple R-squared:  0.1107, Adjusted R-squared:  -0.01636
## F-statistic: 0.8712 on 3 and 21 DF,  p-value: 0.4716
```

```
##
## Call:
## lm(formula = MADRS_avsluttet ~ tNAA.3 + kjønn + alder_ved_start,
##     data = MADRS_data.1, na.action = na.omit)
##
## Residuals:
##      Min       1Q   Median       3Q      Max
## -14.8382  -6.4508   0.6202   6.4616  13.8853
##
## Coefficients:
##                 Estimate Std. Error t value Pr(>|t|)
## (Intercept)      54.8549    28.7774   1.906   0.0711 .
## tNAA.3          -21.8878    19.0732  -1.148   0.2647
## kjønn             0.5087     3.7663   0.135   0.8939
## alder_ved_start  -0.2538     0.1447  -1.754   0.0948 .
## ---
## Signif. codes:  0 '***' 0.001 '**' 0.01 '*' 0.05 '.' 0.1 ' ' 1
##
## Residual standard error: 8.473 on 20 degrees of freedom
##   (7 observations deleted due to missingness)
## Multiple R-squared:  0.1496, Adjusted R-squared:  0.022
## F-statistic: 1.172 on 3 and 20 DF,  p-value: 0.3451
```

```
##
## Call:
## lm(formula = delta_MADRS ~ delta_tNAA + kjønn + alder_ved_start,
##     data = MADRS_data.1, na.action = na.omit)
##
## Residuals:
##      Min       1Q   Median       3Q      Max
## -19.3570  -8.8581   0.5625   7.3121  20.0805
##
## Coefficients:
##                 Estimate Std. Error t value Pr(>|t|)
## (Intercept)     -16.1924     7.5446  -2.146   0.0466 *
## delta_tNAA      -37.0114    32.1504  -1.151   0.2656
## kjønn            -0.6810     5.1016  -0.133   0.8954
## alder_ved_start  -0.1168     0.1667  -0.700   0.4932
## ---
## Signif. codes:  0 '***' 0.001 '**' 0.01 '*' 0.05 '.' 0.1 ' ' 1
##
## Residual standard error: 10.87 on 17 degrees of freedom
##   (10 observations deleted due to missingness)
## Multiple R-squared:  0.09545,    Adjusted R-squared:  -0.06418
## F-statistic: 0.598 on 3 and 17 DF,  p-value: 0.625
```

tNAA and EMQ

tNAA/H2O

```
##
## Call:
## lm(formula = EMQ_før_samlescore ~ NAA.1 + kjønn + alder_ved_start,
##     data = EMQ_data, na.action = na.omit)
##
## Residuals:
##     Min      1Q  Median      3Q     Max
## -55.977 -25.610  -6.504  19.165  74.080
##
## Coefficients:
##                  Estimate Std. Error t value Pr(>|t|)
## (Intercept)     -30.31751  145.18050  -0.209    0.837
## NAA.1            15.76341   14.91152   1.057    0.302
## kjønn            -6.03552   16.49278  -0.366    0.718
## alder_ved_start   0.03565    0.52905   0.067    0.947
##
## Residual standard error: 37.17 on 21 degrees of freedom
##   (8 observations deleted due to missingness)
## Multiple R-squared:  0.07131,    Adjusted R-squared:  -0.06136
## F-statistic: 0.5375 on 3 and 21 DF,  p-value: 0.6617
```

```
##
## Call:
## lm(formula = delta_EMQ ~ delta_NAA + kjønn + alder_ved_start,
##     data = EMQ_data, na.action = na.omit)
##
## Residuals:
##     Min      1Q  Median      3Q     Max
## -48.007 -21.000  -9.141  20.851  65.866
##
## Coefficients:
##                 Estimate Std. Error t value Pr(>|t|)
## (Intercept)      25.4002    24.2977   1.045    0.310
## delta_NAA        15.4987    17.4455   0.888    0.387
## kjønn            -6.9170    17.1201  -0.404    0.691
## alder_ved_start  -0.4630     0.4966  -0.932    0.364
##
## Residual standard error: 35.62 on 17 degrees of freedom
##   (12 observations deleted due to missingness)
## Multiple R-squared:  0.1121, Adjusted R-squared:  -0.04456
## F-statistic: 0.7156 on 3 and 17 DF,  p-value: 0.5561
```

```
##
## Call:
## lm(formula = EMQ_etter_samlescore ~ NAA.3 + kjønn + alder_ved_start,
##     data = EMQ_data, na.action = na.omit)
##
## Residuals:
##    Min     1Q Median     3Q    Max
## -40.76 -20.70  -7.92  14.71  56.17
##
## Coefficients:
##                 Estimate Std. Error t value Pr(>|t|)
## (Intercept)     -42.0287    99.5284  -0.422   0.6773
## NAA.3            20.3682    10.4460   1.950   0.0654 .
## kjønn           -10.8416    12.6649  -0.856   0.4021
## alder_ved_start  -0.5046     0.4087  -1.235   0.2313
## ---
## Signif. codes:  0 '***' 0.001 '**' 0.01 '*' 0.05 '.' 0.1 ' ' 1
##
## Residual standard error: 29.09 on 20 degrees of freedom
##   (9 observations deleted due to missingness)
## Multiple R-squared:  0.321,  Adjusted R-squared:  0.2191
## F-statistic: 3.151 on 3 and 20 DF,  p-value: 0.04758
```

tNAA/tCr

```
##
## Call:
## lm(formula = EMQ_før_samlescore ~ tNAA.1 + kjønn + alder_ved_start,
##     data = EMQ_data, na.action = na.omit)
##
## Residuals:
##     Min      1Q  Median      3Q     Max
## -69.095 -18.541  -7.707  30.719  72.806
##
## Coefficients:
##                 Estimate Std. Error t value Pr(>|t|)
## (Intercept)     249.5629   110.3151   2.262   0.0344 *
## tNAA.1          -85.8856    68.8383  -1.248   0.2259
## kjønn            -7.4059    15.5723  -0.476   0.6393
## alder_ved_start  -0.2252     0.5867  -0.384   0.7050
## ---
## Signif. codes:  0 '***' 0.001 '**' 0.01 '*' 0.05 '.' 0.1 ' ' 1
##
## Residual standard error: 35.63 on 21 degrees of freedom
##   (6 observations deleted due to missingness)
## Multiple R-squared:  0.08468,    Adjusted R-squared:  -0.04609
## F-statistic: 0.6476 on 3 and 21 DF,  p-value: 0.5932
```

```
##
## Call:
## lm(formula = EMQ_etter_samlescore ~ tNAA.3 + kjønn + alder_ved_start,
##     data = EMQ_data, na.action = na.omit)
##
## Residuals:
##     Min      1Q  Median      3Q     Max
## -31.601 -17.937  -7.460   7.086  55.066
##
## Coefficients:
##                  Estimate Std. Error t value Pr(>|t|)
## (Intercept)     -34.94807   92.75975  -0.377   0.7105
## tNAA.3          114.70756   61.36326   1.869   0.0771 .
## kjønn           -18.00504   12.33412  -1.460   0.1607
## alder_ved_start   0.07506    0.46565   0.161   0.8736
## ---
## Signif. codes:  0 '***' 0.001 '**' 0.01 '*' 0.05 '.' 0.1 ' ' 1
##
## Residual standard error: 27.24 on 19 degrees of freedom
##   (8 observations deleted due to missingness)
## Multiple R-squared:  0.2359, Adjusted R-squared:  0.1152
## F-statistic: 1.955 on 3 and 19 DF,  p-value: 0.155
```

```
##
## Call:
## lm(formula = delta_EMQ ~ delta_tNAA + kjønn + alder_ved_start,
##     data = EMQ_data, na.action = na.omit)
##
## Residuals:
##     Min      1Q  Median      3Q     Max
## -56.499 -17.411   0.956  20.435  64.965
##
## Coefficients:
##                 Estimate Std. Error t value Pr(>|t|)
## (Intercept)      14.3407    26.1518   0.548    0.591
## delta_tNAA     -110.7528   130.6626  -0.848    0.409
## kjønn             0.1112    18.5381   0.006    0.995
## alder_ved_start  -0.5166     0.5663  -0.912    0.375
##
## Residual standard error: 36.63 on 16 degrees of freedom
##   (11 observations deleted due to missingness)
## Multiple R-squared:  0.08782,    Adjusted R-squared:  -0.08321
## F-statistic: 0.5135 on 3 and 16 DF,  p-value: 0.6788
```

tCho

tCho and EMQ

tCho/tCr

```
##
## Call:
## lm(formula = EMQ_før_samlescore ~ tCho.1 + kjønn + alder_ved_start,
##     data = EMQ_data, na.action = na.omit)
##
## Residuals:
##    Min     1Q Median     3Q    Max
## -69.48 -19.23 -11.42  18.72  73.43
##
## Coefficients:
##                 Estimate Std. Error t value Pr(>|t|)
## (Intercept)     139.8753    58.5387   2.389   0.0263 *
## tCho.1          -92.3200   198.0157  -0.466   0.6459
## kjønn            -6.7348    17.0989  -0.394   0.6976
## alder_ved_start   0.1253     0.5377   0.233   0.8180
## ---
## Signif. codes:  0 '***' 0.001 '**' 0.01 '*' 0.05 '.' 0.1 ' ' 1
##
## Residual standard error: 36.73 on 21 degrees of freedom
##   (6 observations deleted due to missingness)
## Multiple R-squared:  0.0269, Adjusted R-squared:  -0.1121
## F-statistic: 0.1935 on 3 and 21 DF,  p-value: 0.8996
```

```
##
## Call:
## lm(formula = delta_EMQ ~ delta_tCho_tCr + kjønn + alder_ved_start,
##     data = EMQ_data, na.action = na.omit)
##
## Residuals:
##     Min      1Q  Median      3Q     Max
## -61.919 -15.602   3.412  18.339  54.907
##
## Coefficients:
##                 Estimate Std. Error t value Pr(>|t|)
## (Intercept)       5.1932    25.4229   0.204    0.841
## delta_tCho_tCr  656.5670   387.2318   1.696    0.109
## kjønn            -1.3746    16.6270  -0.083    0.935
## alder_ved_start  -0.1318     0.5427  -0.243    0.811
##
## Residual standard error: 34.47 on 16 degrees of freedom
##   (11 observations deleted due to missingness)
## Multiple R-squared:  0.192,  Adjusted R-squared:  0.04054
## F-statistic: 1.268 on 3 and 16 DF,  p-value: 0.319
```

tCho/H2O

```
##
## Call:
## lm(formula = EMQ_før_samlescore ~ tCho.1 + kjønn + alder_ved_start,
##     data = EMQ_TC_data, na.action = na.omit)
##
## Residuals:
##     Min      1Q  Median      3Q     Max
## -64.388 -17.485  -9.262  26.221  69.697
##
## Coefficients:
##                  Estimate Std. Error t value Pr(>|t|)
## (Intercept)      82.87133   52.17139   1.588    0.127
## tCho.1           12.98388   19.04625   0.682    0.503
## kjønn           -12.17749   16.30411  -0.747    0.463
## alder_ved_start  -0.06609    0.59431  -0.111    0.913
##
## Residual standard error: 36.52 on 21 degrees of freedom
##   (6 observations deleted due to missingness)
## Multiple R-squared:  0.03811,    Adjusted R-squared:  -0.0993
## F-statistic: 0.2774 on 3 and 21 DF,  p-value: 0.8411
```

```
##
## Call:
## lm(formula = delta_EMQ ~ delta_tCho + kjønn + alder_ved_start,
##     data = EMQ_TC_data, na.action = na.omit)
##
## Residuals:
##     Min      1Q  Median      3Q     Max
## -53.292 -17.215  -6.855  19.463  58.166
##
## Coefficients:
##                 Estimate Std. Error t value Pr(>|t|)
## (Intercept)      13.2450    22.3671   0.592   0.5620
## delta_tCho       55.0981    21.8031   2.527   0.0224 *
## kjønn             2.6219    15.4384   0.170   0.8673
## alder_ved_start  -0.3823     0.4760  -0.803   0.4337
## ---
## Signif. codes:  0 '***' 0.001 '**' 0.01 '*' 0.05 '.' 0.1 ' ' 1
##
## Residual standard error: 31.65 on 16 degrees of freedom
##   (11 observations deleted due to missingness)
## Multiple R-squared:  0.3188, Adjusted R-squared:  0.191
## F-statistic: 2.496 on 3 and 16 DF,  p-value: 0.09693
```

mI

mI and MADRS

mI/tCr

```
##
## Call:
## lm(formula = MADRS_0 ~ mI.1 + kjønn + alder_ved_start, data = MADRS_data.1,
##     na.action = na.omit)
##
## Residuals:
##     Min      1Q  Median      3Q     Max
## -7.1996 -3.7499 -0.4636  4.4745 10.1286
##
## Coefficients:
##                 Estimate Std. Error t value Pr(>|t|)
## (Intercept)     38.26417    9.07242   4.218 0.000386 ***
## mI.1            -1.88996   13.31841  -0.142 0.888507
## kjønn           -1.75008    2.27693  -0.769 0.450681
## alder_ved_start -0.04548    0.07751  -0.587 0.563578
## ---
## Signif. codes:  0 '***' 0.001 '**' 0.01 '*' 0.05 '.' 0.1 ' ' 1
##
## Residual standard error: 5.219 on 21 degrees of freedom
##   (6 observations deleted due to missingness)
## Multiple R-squared:  0.07272,    Adjusted R-squared:  -0.05975
## F-statistic: 0.5489 on 3 and 21 DF,  p-value: 0.6544
```

```
##
## Call:
## lm(formula = MADRS_avsluttet ~ mI.3 + kjønn + alder_ved_start,
##     data = MADRS_data.1, na.action = na.omit)
##
## Residuals:
##      Min       1Q   Median       3Q      Max
## -11.3324  -6.2842   0.1692   4.6505  15.5705
##
## Coefficients:
##                 Estimate Std. Error t value Pr(>|t|)
## (Intercept)      45.4957    15.7712   2.885  0.00916 **
## mI.3            -36.5262    23.3775  -1.562  0.13387
## kjønn            -1.3375     3.5256  -0.379  0.70842
## alder_ved_start  -0.1233     0.1252  -0.985  0.33638
## ---
## Signif. codes:  0 '***' 0.001 '**' 0.01 '*' 0.05 '.' 0.1 ' ' 1
##
## Residual standard error: 8.258 on 20 degrees of freedom
##   (7 observations deleted due to missingness)
## Multiple R-squared:  0.1922, Adjusted R-squared:  0.071
## F-statistic: 1.586 on 3 and 20 DF,  p-value: 0.2241
```

```
##
## Call:
## lm(formula = delta_MADRS ~ mI.3 + kjønn + alder_ved_start, data = MADRS_data.1,
##     na.action = na.omit)
##
## Residuals:
##     Min      1Q  Median      3Q     Max
## -20.341  -5.796  -1.884   7.713  13.997
##
## Coefficients:
##                 Estimate Std. Error t value Pr(>|t|)
## (Intercept)      11.25491   19.54933   0.576    0.571
## mI.3            -43.64875   28.97773  -1.506    0.148
## kjønn            -1.83820    4.37015  -0.421    0.679
## alder_ved_start  -0.01309    0.15513  -0.084    0.934
##
## Residual standard error: 10.24 on 20 degrees of freedom
##   (7 observations deleted due to missingness)
## Multiple R-squared:  0.1164, Adjusted R-squared:  -0.01609
## F-statistic: 0.8786 on 3 and 20 DF,  p-value: 0.4688
```

mI/H2O

```
##
## Call:
## lm(formula = MADRS_0 ~ Ins.1 + kjønn + alder_ved_start, data = MADRS_data.1,
##     na.action = na.omit)
##
## Residuals:
##    Min     1Q Median     3Q    Max
## -7.877 -3.738 -1.105  4.458 10.516
##
## Coefficients:
##                 Estimate Std. Error t value Pr(>|t|)
## (Intercept)     34.02181    7.48684   4.544 0.000177 ***
## Ins.1            0.51994    1.15107   0.452 0.656113
## kjønn           -1.72651    2.26007  -0.764 0.453415
## alder_ved_start -0.06831    0.08893  -0.768 0.450927
## ---
## Signif. codes:  0 '***' 0.001 '**' 0.01 '*' 0.05 '.' 0.1 ' ' 1
##
## Residual standard error: 5.197 on 21 degrees of freedom
##   (6 observations deleted due to missingness)
## Multiple R-squared:  0.08076,    Adjusted R-squared:  -0.05056
## F-statistic: 0.615 on 3 and 21 DF,  p-value: 0.6129
```

```
##
## Call:
## lm(formula = MADRS_avsluttet ~ Ins.3 + kjønn + alder_ved_start,
##     data = MADRS_data.1, na.action = na.omit)
##
## Residuals:
##      Min       1Q   Median       3Q      Max
## -11.3548  -6.2319   0.0484   4.2201  15.1629
##
## Coefficients:
##                 Estimate Std. Error t value Pr(>|t|)
## (Intercept)     37.91553   11.09186   3.418  0.00272 **
## Ins.3           -2.62432    1.62407  -1.616  0.12178
## kjønn           -2.34348    3.62693  -0.646  0.52554
## alder_ved_start -0.04691    0.14297  -0.328  0.74627
## ---
## Signif. codes:  0 '***' 0.001 '**' 0.01 '*' 0.05 '.' 0.1 ' ' 1
##
## Residual standard error: 8.227 on 20 degrees of freedom
##   (7 observations deleted due to missingness)
## Multiple R-squared:  0.1982, Adjusted R-squared:  0.07798
## F-statistic: 1.648 on 3 and 20 DF,  p-value: 0.2101
```

```
##
## Call:
## lm(formula = delta_MADRS ~ Ins.3 + kjønn + alder_ved_start, data = MADRS_data.1,
##     na.action = na.omit)
##
## Residuals:
##     Min      1Q  Median      3Q     Max
## -19.747  -6.310  -1.465   7.283  14.309
##
## Coefficients:
##                 Estimate Std. Error t value Pr(>|t|)
## (Intercept)      2.49271   13.72557   0.182    0.858
## Ins.3           -3.18654    2.00970  -1.586    0.129
## kjønn           -3.07067    4.48813  -0.684    0.502
## alder_ved_start  0.08054    0.17692   0.455    0.654
##
## Residual standard error: 10.18 on 20 degrees of freedom
##   (7 observations deleted due to missingness)
## Multiple R-squared:  0.1261, Adjusted R-squared:  -0.005028
## F-statistic: 0.9616 on 3 and 20 DF,  p-value: 0.4301
```
